# CSV-Filter: a deep learning-based comprehensive structural variant filtering method for both short and long reads

**DOI:** 10.1093/bioinformatics/btae539

**Published:** 2024-09-06

**Authors:** Zeyu Xia, Weiming Xiang, Qingzhe Wang, Xingze Li, Yilin Li, Junyu Gao, Tao Tang, Canqun Yang, Yingbo Cui

**Affiliations:** College of Computer Science and Technology, National University of Defense Technology, Hunan 410073, P. R. China; College of Computer Science and Electronic Engineering, Hunan University, Hunan 410082, P. R. China; College of Computer Science and Technology, National University of Defense Technology, Hunan 410073, P. R. China; College of Computer Science and Technology, National University of Defense Technology, Hunan 410073, P. R. China; College of Computer Science and Technology, National University of Defense Technology, Hunan 410073, P. R. China; College of Computer Science and Technology, National University of Defense Technology, Hunan 410073, P. R. China; College of Computer Science and Technology, National University of Defense Technology, Hunan 410073, P. R. China; College of Computer Science and Technology, National University of Defense Technology, Hunan 410073, P. R. China; National Supercomputer Center in Tianjin, Tianjin, 300457, P. R. China; Haihe Lab of ITAI, Tianjin, 300457, P. R. China; College of Computer Science and Technology, National University of Defense Technology, Hunan 410073, P. R. China

## Abstract

**Motivation:**

Structural variants (SVs) play an important role in genetic research and precision medicine. As existing SV detection methods usually contain a substantial number of false positive calls, approaches to filter the detection results are needed.

**Results:**

We developed a novel deep learning-based SV filtering tool, CSV-Filter, for both short and long reads. CSV-Filter uses a novel multi-level grayscale image encoding method based on CIGAR strings of the alignment results and employs image augmentation techniques to improve SV feature extraction. CSV-Filter also utilizes self-supervised learning networks for transfer as classification models, and employs mixed-precision operations to accelerate training. The experiments showed that the integration of CSV-Filter with popular SV detection tools could considerably reduce false positive SVs for short and long reads, while maintaining true positive SVs almost unchanged. Compared with DeepSVFilter, a SV filtering tool for short reads, CSV-Filter could recognize more false positive calls and support long reads as an additional feature.

**Availability and implementation:**

https://github.com/xzyschumacher/CSV-Filter

## 1. Introduction

Structural variants (SVs) are a common form of genetic variant and typically refer to structural differences greater than 50 base pairs in genomes, including insertions (INSs), deletions (DELs), duplications, inversions, translocations, etc (Feuk *et al.* 2006). Compared to single nucleotide polymorphisms (SNPs) and small insertions and deletions (INDELs), SVs often have significant impacts on organisms (Garcia-Prieto *et al.* 2022). For example, large INSs or DELs may lead to changes or loss of gene function, resulting in the occurrence of genetic diseases (Sone *et al.* 2019). Replication or amplification of repetitive sequences can alter the copy number of genes, affecting gene expression and function (Chiang *et al.* 2017). Inversion and translocation events can cause rearrangements of chromosomal regions, thereby affecting genome stability and function (C Yuen *et al.* 2017).

The commonly used strategies for detecting SVs can be mainly classified as: Read Depth (RD) based (Klambauer *et al.* 2012), Split Read (SR) based (Ye *et al.* 2009), Discordant Read Pair (RP) based (Chen *et al.* 2009), *de novo* assembly (AS) based (Chen *et al.* 2014), hybrid methods based on multiple operations (Chen *et al.* 2016), and SV signatures for some long-read based callers (Heller and Vingron 2019, Jiang *et al.* 2020).

Current SV detection tools usually yield a substantial number of false positive calls due to the repetitive nature of the human genome, the limitations of existing sequencing technologies and alignment algorithms. To solve this problem, researchers usually filter the results of SV detection to enhance overall accuracy. Existing approaches for SVs filtering involve manual screening with visualization tools such as integrative genomics viewer (IGV) (Robinson *et al.* 2011), svviz (Spies *et al.* 2015), Samplot (Belyeu *et al.* 2021), etc., or the use of heuristic filters with manually selected parameters. These methods are often time-consuming and require expert guidance to determine the appropriate parameters (Liu *et al.* 2021). Therefore, it is necessary to develop an efficient SV filtering tool to filter the detection results.

Recently, deep learning has applied as a new approach for variant calling (Walsh *et al.* 2021). DeepVariant (Poplin *et al.* 2018) utilizes convolutional neural networks (CNNs) and recurrent neural networks (RNNs) to model and forecast sequencing data, enabling precise identification of SNPs and INDELs. Clair3 (Zheng *et al.* 2022) combines deep learning with traditional statistical models to detect single nucleotide variants (SNVs) and INDELs. However, DeepVariant and Clair3 can only detect small-scale variants like SNPs, SNVs, or INDELs. DeepSVFilter (Liu *et al.* 2021) is a deep learning-based SV filtering tool. It maps input genomic data into images through feature extraction and subsequently employs CNNs and RNNs to learn the mapping relationship from features to SVs. This process enables the filtering of potential SV candidates, thereby reducing false positive SV calls, but DeepSVFilter can only filter results generated by SV detection tools for short reads.

The third-generation sequencing is characterized by long read length and high error rate (Jackman *et al.* 2018). The long read length facilitates the detection of large-scale genomic variants, while the high error rate increases the risk of generating false positive calls during variant detection, making it necessary to develop specialized SV detection algorithms for long reads. Some SV detection tools for long reads have been developed, including PBSV (Pacific Biosciences 2021), Sniffles2 (Sedlazeck *et al.* 2018), SVIM (Heller and Vingron 2019), cuteSV (Jiang *et al.* 2020), SVision (Lin *et al.* 2022), SVcnn (Zheng and Shang 2023), cnnLSV (Ma *et al.* 2023), etc. Although these third-generation SV detection tools have made great strides, they still suffer from the large number of false positive calls (Kosugi *et al.* 2019). The SV detection tools for long reads also require proper filtering methods.

In this article, we developed CSV-Filter, a deep learning-based SV filtering tool for both short reads and long reads. CSV-Filter uses a novel multi-level grayscale image encoding method based on the CIGAR string in the sequence alignment information, which ensures the robust applicability to both short and long reads. We redefined the transfer learning preprocessing layers and applied image augmentation to the generated images. CSV-Filter also employs transfer learning of fine-tuning (Szegedy *et al.* 2016) for a self-supervised pre-trained model, which boosts the model’s accuracy and generalization ability, and significantly reduces the need for large amounts of annotated data by traditional CNN models for supervised learning. Lastly, CSV-Filter utilizes mixed-precision operations to accelerate the training process and save the GPU memory footprint. Experiments show that the integration of CSV-Filter with popular SV detection tools can significantly reduce false positive SV calls for both short reads and long reads.

## 2. Materials and methods

**Figure 1.**
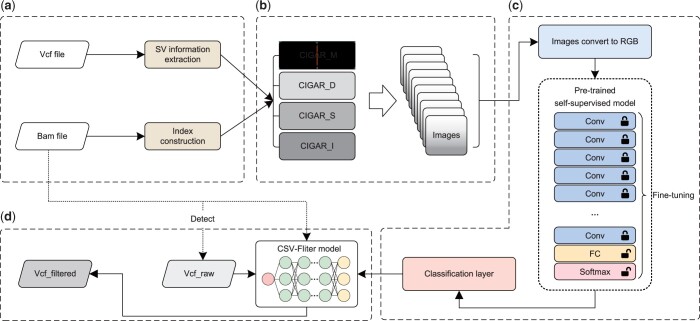
The workflow of CSV-Filter. **a**, SV information extraction and alignment file index construction. **b**, Multi-level grayscale image encoding based on CIGAR strings. **c**, Model training and SVs classification. **d**, Filtering for SV detection results.

The workflow of CSV-Filter is illustrated in Fig. 1. CSV-Filter first extracts SV information from a high-confidence SV call set and constructs an index for the alignment file (Fig. 1a). This step involves obtaining SV sites and their corresponding information, while the alignment file index construction ensures the retrieval of alignment information in subsequent operations. Subsequently, CSV-Filter selects the reads within each SV region and encodes a multi-level grayscale image for each SV site based on the CIGAR strings of the selected reads (Fig. 1b). The generated images are then transformed to meet the input requirements of the model through pre-processing layers in transfer learning (Fig. 1c). During training, CSV-Filter employs a pre-trained self-supervised learning model and classify the corresponding images into different SV types based on the training results. Finally, CSV-Filter utilizes the trained model to filter SV detection results, and output the filtered variants (Fig. 1d).

### 2.1 Multi-level grayscale image encoding based on CIGAR strings

The main challenge in utilizing deep learning for variant filtering lies in encoding sequence information into image representations while preserving the original SV information as much as possible. To address this challenge, we proposed a multi-level grayscale image encoding method based on CIGAR strings. The utilization of CIGAR strings offers three distinct advantages: 1) CIGAR strings are universally present in alignment files by both short reads and long reads, making them highly versatile for diverse sequencing technologies. 2) CIGAR format defines nine types of operations to represent alignment results: M (MATCH), I (INSERT), D (DELETE), N (SKIP), S (SOFT CLIP), H (HARD CLIP), P (PAD), = (SEQUENCE MATCH), and X (SEQUENCE MISMATCH) (Danecek *et al.* 2021), which are applicable to various alignment scenarios. 3) CIGAR strings contain length information that represents the relative position between the aligned reads and reference genome, including the number of inserted or deleted bases and other variant features.

**Figure 2.**
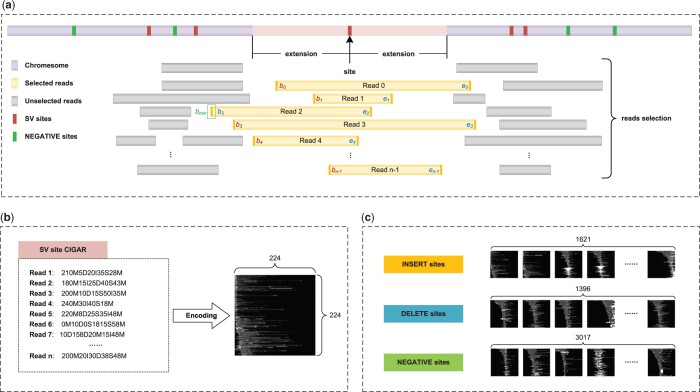
Multi-level grayscale CIGAR strings image encoding process. **a**, Site position extension and overlap reads selection. **b**, An example for one INSERT site image encoding of Chromosome 1. **c**, Image encoding results with different SVs of Chromosome 1.

Figure 2 shows the image encoding process in CSV-Filter, which can be mainly divided into three steps: 1) sites locating, 2) reads selection, and 3) images encoding.

#### 2.1.1 Sites locating

CSV-Filter encodes one image for each SV site. These SV sites are extracted from high-confidence SV call set. As the high-confidence SV call set does not contain negative samples required by model training, we need to generate an appropriate number of negative samples to train and evaluate the model.

By analyzing the distribution of SV regions, we found that the lengths of SVs follow a Poisson distribution (Xiang *et al.* 2022). We calculated the mean and variance of SVs, and its harmonic mean was computed as the mean and variance for the negative samples. The negative samples were generated using the probability density function of the Poisson distribution, as shown in equation 1.

(1) $$\begin{array}{c} \begin{array}{c} \lambda_{\mathrm{neg}}=\lambda_{sv},\\ P(X=k)=\frac{e^{-\lambda_{\mathrm{neg}}}\cdot \lambda_{\mathrm{neg}}^{k}}{k!},(k=0,1,2,\ldots ) \end{array} \end{array}$$

where $\lambda_{sv}$ and $\lambda_{\mathrm{neg}}$ represent the mean and variance of SVs and negative samples, respectively.

CSV-Filter generates negative samples iteratively. The generated sample will be dropped and regenerated if it overlaps more than half with the adjacent SVs. CSV-Filter repeats this process until a sufficient number of negative samples are obtained. After the iterations completed, CSV-Filter normalizes the outputs to guarantee that the generated samples adhere to the acceptable range. The details of this process are provided in Algorithm S1.

#### 2.1.2 Reads selection

Once all SV sites are located, CSV-Filter will select corresponding reads for each site. Figure 2a illustrates this process. CSV-Filter extends forward and backward from each site by a certain distance, and selects the reads that overlap with the extended regions.

#### 2.1.3 Images encoding

CSV-Filter encodes images based on the CIGAR strings included in the alignment information of selected reads. We collected a large number of alignment results from several major genome projects and made statistics on the CIGAR operations. The statistics revealed that the operations “M,” “I,” “D,” and “S” together occupied a very high proportion (over 98%). Supplementary Figure S2 and Supplementary Table S1 show the proportion of CIGAR operations in the alignment files. Hence, we chose the most representative “M,” “I,” “D,” and “S” operations to encode image, which can not only enhance model accuracy and data processing efficiency but also mitigate the risk of overfitting and unnecessary data redundancy.

CSV-Filter encodes one image for each SV site. Figure 2b describes the process of image encoding. It mainly consists of five steps: Firstly, CSV-Filter iterates through the selected reads to identify the minimum starting position, $b_{\min}$. This step ensures that the encoded image contains the CIGAR information of all related reads. Next, CSV-Filter calculates the offset $b_{i}-b_{\min}$ between the current read and the minimum starting position $b_{\min}$ to determine the *x*-axis range $\left(b_{i}-b_{\min},e_{i}\right)$ of the encoded image, where $b_{i}$ and $e_{i}$ represent the starting and ending positions of the current read, respectively. Then, CSV-Filter uses different grayscale values in the range (0, 255) to represent the four operations “M,” “I,” “D,” and “S,” based on the CIGAR strings values of the current read. For offset distances and operations like “N,” “P,” “H,” “=,” and “X,” the corresponding grayscale values are set to 0. Following this, CSV-Filter iterates through all selected reads to generate the raw image. Finally, the raw image is normalized by stretching/compressing its *x*-axis and *y*-axis lengths to 224. This normalization ensures that the encoded images conform to the input dimensions required for the subsequent transfer learning phase. The detailed process of image encoding is provided in Algorithm S2. Figure 2c shows the images generated from Chromosome 1 of Homo Sapiens, including 1621 INSs 1396 DELs, and 3017 negative sites.

### 2.2 Transfer learning-based self-supervised learning model training

In the field of deep learning, training a new model from scratch is an extremely time-consuming and computationally demanding process. Moreover, such models often encounter challenges such as high data requirements, poor generalization performance, and catastrophic parameter initialization. To address these difficulties, we employed transfer learning techniques to train CSV-Filter. Transfer learning is a deep learning approach that leverages knowledge acquired from a source domain to help the learning in a target domain. In image encoding process, the CIGAR strings of reads are encoded into images. By employing transfer learning, a pre-trained model is utilized for feature extraction and discrimination of these encoded images. This training method will effectively address data scarcity issues within variant calling, improve model generalization capabilities, and reduce training time. The implementation of transfer learning primarily involves three steps: RGB conversion, fine-tuning, and classification.

#### 2.2.1 RGB conversion

The pre-processing layer in transfer learning provides appropriate input data to facilitate effective knowledge transfer and model training. We redefined the pre-processing layer in CSV-Filter, which encompasses two aspects. Initially, it adjusts the encoded images to meet the requirements of pre-trained models used in transfer learning, thereby enhancing the model’s ability to extract SV features. Given that the encoded images are grayscale and sized 224 × 224, CSV-Filter converts the image data to the Python Imaging Library (PIL) format and transforms the input image to RGB mode, ensuring compliance with the pre-trained model’s requirements. Subsequently, it applies random color jitter transformations to the converted RGB images to increase data diversity and mitigate data imbalance issues. At the same time, we normalize the image data to improve the model’s stability and generalization ability, ensuring a consistent scale and distribution of the input data. These steps boost the model’s performance and facilitate better compatibility with pre-trained models.

#### 2.2.2 Fine-tuning

In traditional transfer learning, the training is typically conducted with two separate components: The feature extractor and the classifier. Fine-tuning improves traditional transfer learning by training not only the classifier but also the entire model, making it more flexible and comprehensive.

CSV-Filter employs fine-tuning to further train a pre-trained self-supervised learning model for SV filtering. Fine-tuning consists of two main steps: Pre-training and fine-tuning. Pre-training utilizes self-supervised learning, an unsupervised learning method that designs tasks for the model to generate labels or targets from unlabeled data, thereby learning useful representations or features. Compared to conventional supervised learning, self-supervised learning does not require manual annotation and can leverage unlabeled data to address these challenges, thus overcoming the dependency on a large amount of labeled data. Self-supervised learning also exhibits strong generalization ability. By conducting self-supervised learning on a large-scale unlabeled dataset, the model can learn generic feature representations that can be transferred and applied across various tasks and domains. This enables the model to perform well and exhibit better generalization capabilities when facing tasks with limited labeled data.

We employed Variance-Invariance-Covariance Regularization (VICReg) (Bardes *et al.* 2021) to regularize the output representations of the model. VICReg can address potential collapse issues during model training through three regularization terms: Variance, covariance, and invariance. Variance regularization maintains the variance of each embedding dimension above a certain threshold, preventing all inputs from mapping to the same vector. Covariance regularization reduces the covariance between pairs of embedding variables to near 0, decorrelating the variables and preventing information redundancy. Invariance regularization minimizes the distance between the embedding vectors of different views of the same image. During the fine-tuning step, we introduce negative samples to enhance the discriminative capability of the self-supervised model. Additionally, the inclusion of negative samples prevents all inputs from mapping to the same embedding during the training phase, further mitigating the risk of representation collapse.

After pre-training, the pre-trained model is further trained to adapt to the task of SV filtering. The specific steps include: importing the pre-trained model, freezing certain layers of the network, adjusting the learning rate appropriately, retraining and fine-tuning the model using the encoded image data, and iteratively optimizing the model. Through fine-tuning, the model is able to leverage the generic features learned during the pre-training step and make specific adjustments for the task of SV filtering, thereby improving the overall performance of the model.

#### 2.2.3 Classification

After each training iteration, the classification layer in transfer learning utilizes the extracted features from the trained model to perform classification of SVs based on the pre-defined labels. It consists of attention fully connected units, fully connected units, and fully connected classification units. The attention fully connected unit is composed of three sequential operations: Attention operation, fully connected operation, and ReLU activation operation. The fully connected units include a fully connected operation and a ReLU activation operation in sequential order. The fully connected classification units include a fully connected operation and Softmax operation. We combined two attention fully connected units and one fully connected unit as a one-dimensional attention residual module to accomplish feature extraction. After the above operations, the extracted features are fed into the fully connected classification units to obtain probabilities corresponding to each SV type. The classification result of the SV is determined by selecting the SV type with the highest probability value. The details of classification layer are provided in Supplementary Figure S1 and Supplementary Table S2.

Additionally, CSV-Filter adopts mixed precision operations for model training to address the issues of long training times and high GPU memory usage. For computationally intensive operations such as matrix multiplication and convolution, CSV-Filter employs low precision, thereby reducing memory usage and computational workload, and accelerating the training and inference speed. For critical steps involving gradient updates and parameter updates, which are sensitive to numerical precision, CSV-Filter still employs high precision in order to ensure the accuracy and stability of the model. Overall, adopting mixed precision reduces CSV-Filter’s runtime and GPU memory usage by approximately 45% and 42%, respectively, with the model’s overall accuracy almost unchanged. Experimental details are shown in Supplementary Figures S4 and S5.

### 2.3 Filtering SV detection results

Once the training is complete, CSV-Filter can utilize the trained model to filter the SV detection results. During this process, CSV-Filter is capable of processing the SV calls generated from both short reads and long reads. Figure 1d illustrates the main process of filtering. Initially, the SV detection tool analyses alignment sequences and generates the raw SV calls. Next, CSV-Filter extracts the corresponding SV information based on these raw SV calls. Subsequently, CSV-Filter employs the same approach to encode the SV information into images. Finally, CSV-Filter applies the trained model to filter the generated images and identify false positive SV calls.

## 3. Experiments and results

### 3.1 Datasets and experimental configuration

In this study, we used two samples, HG002 and NA12878, from the NIST’s Genome in a Bottle (GIAB) project (Zook *et al.* 2014) to evaluate the performance of CSV-Filter. The Tier 1 benchmark SV callset covers 2.51 Gbp and includes 4,199 deletions and 5,442 insertions in defined high-confidence HG002 region (Zook *et al.* 2020). Raw PacBio CLR, HiFi, and ONT reads were aligned to the GRCh37 using minimap2 (v2.28), pbmm2 (v1.13.1), and NGMLR (v0.2.7). Raw Illumina reads were aligned to the hs37d5 reference using BWA-MEM (Li 2013) (v0.7.17-r1188). The sample NA12878 gold standard SV set includes 3,789 deletions and 5,815 insertions. Raw PacBio CLR and Illumina reads were aligned to hg19 and GRCh38DH using BLASR v1.3.2 and BWA-MEM, respectively. The details of datasets are provided in the Supplementary data.

In the experiments, we used the sample HG002 PacBio HiFi dataset for model training and accuracy assessment. We randomly selected 80% of the data as the training set and the remaining 20% as the validation and test sets. In the evaluation of CSV-Filter’s filtering performance, we first tested the filtering performance of CSV-Filter on long reads. Subsequently, we compared the filtering performance of CSV-Filter with DeepSVFilter on short reads. We chose a range of quality metrics in deep learning to evaluate the performance of the model. These metrics include the Receiver Operating Characteristic (ROC), accuracy, precision, recall, F1 score, etc. The details of these metrics are provided in the Supplementary data.

CSV-Filter is implemented based on the PyTorch framework. We trained our model using the Adam optimizer (Kingma and Ba 2014). The parameters used by read alignment, SV detection, and validation tools in the experiments can be found in Supplementary data. The configuration of the server used is provided in Supplementary Table S3.

### 3.2 Model performance in CNN and self-supervised learning models

In order to demonstrate the discriminative accuracy of CSV-Filter, we conducted validation using 5 CNN models and 4 self-supervised models. The 5 CNN models used were MobileNet v2, ResNet34, ResNet50, ResNet50(x2) and ResNet200(x2). MobileNet v2 and ResNet models are based on the PyTorch framework and are pre-trained using the ImageNet dataset (Deng *et al.* 2009). With the powerful feature discriminative capabilities of the ImageNet pre-trained models, the trained models achieved classification of SVs. We first compared the discriminative performance of different types of models. Then, we discussed the impact of different depths and widths on the discriminative performance within ResNet models. Finally, we compared the impact of self-supervised learning on model accuracy. The details of the nine models and their training process are provided in Supplementary Table S4 and Supplementary Figures S6–S11.

To evaluate the performance of CSV-Filter, we computed the metrics separately for precision, recall, and F1 score, and then obtained the macro-averaged values across them as the evaluation results in CNN models. To comprehensively assess the discriminative performance, we compared the F1 scores for each SV. The results are presented in Supplementary Tables S5–S7. From the results, CSV-Filter achieved its best performance with the ResNet50(x2) model. The model’s accuracy reached 94.05%. Compared to the CNN models, CSV-Filter demonstrated performance improvements after incorporating self-supervised training. Specifically, the ResNet50(x2) model achieved a performance gain of 0.89%, and the F1 score of INS, DEL, and NEG (negative samples) reaches 96.28%, 92.81%, and 95.06% respectively. This result indicates that the self-supervised learning models with VICReg regularization exhibit stronger generalization capabilities and robustness, enabling better feature discrimination.

**Figure 3.**
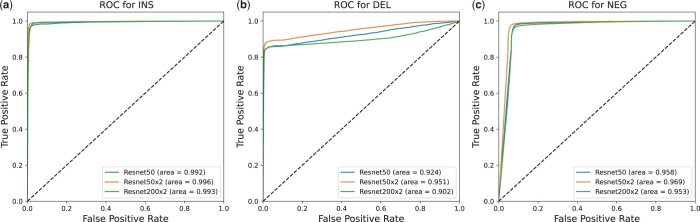
ROC curve of self-supervised learning models ResNet50, ResNet50x2, and ResNet200x2. **a**, The ROC curves for insertion discrimination. **b**, The ROC curves for deletion discrimination. **c**, The ROC curves for negative samples discrimination.

Figure 3 depicts the discriminative performance of the three self-supervised learning models. The ROC-AUC values for INS discrimination reached as high as 0.996, and each model’s ROC-AUC values exceeded 0.9 for all three discriminations. The performance of the models further improved when the model width doubled (Supplementary Table S6). As more parameters were added, the performance declined, even slightly falling below the level of the original ResNet50 model. This indicates that increasing the model width allows the model to capture more discriminative features, thereby improving discriminative performance. With the addition of more parameters, the model may overfit during discrimination, leading to a decrease in accuracy. Considering all factors, the ResNet50(x2) model achieved a more balanced performance.

### 3.3 Filtering performance for long reads

To evaluate the filtering performance of CSV-Filter, we initially employed widely adopted SV detection tools, namely cuteSV (Jiang *et al.* 2020) (v2.0.3), PBSV (Pacific Biosciences 2021) (v2.9.0), Sniffles2 (Sedlazeck *et al.* 2018) (v2.0.7), SVIM (Heller and Vingron 2019) (v2.0.0), and SVision (Lin *et al.* 2022) (v1.3.8), to detect SVs from the sample HG002’s PacBio CLR, PacBio HiFi, and ONT reads. Subsequently, we employed Truvari (English *et al.* 2022) (v3.5.0, with parameters *p *=* *0, *P *=* *0.5, *r *=* *500, *O *=* *0) to validate the SV detection results before and after applying CSV-Filter. Based on the results, we calculated the corresponding recall, precision, and F1 score. The detailed configuration and explanations can be found in Supplementary data.

Table 1 shows the performance of CSV-Filter in filtering long reads. It can be observed that the precisions increase, while the recalls do not significantly decrease for PacBio CLR, PacBio HiFi, and ONT reads before and after filtering. CSV-Filter reduces false positives while maintaining the number of true positives. Notably, for PBSV and Sniffles2 on PacBio CLR reads and PBSV on PacBio HiFi reads, CSV-Filter improved the precision by 6.23%, 4.39%, and 11.05%, respectively, while keeping the recall almost unchanged.

**Table 1.** The filtering performance of CSV-Filter for HG002 long reads.

Platform	SV caller	Without filtering			With filtering		
		Recall^a^ (%)	Precision^b^ (%)	F1 score (%)	Recall (%)	Precision (%)	F1 score (%)
PacBio CLR	cuteSV	95.68	92.29	**93.96**	95.60	92.30	93.92
	PBSV	87.55	86.84	87.19	87.48	93.07	**90.19**
	Sniffles2	94.71	73.09	82.51	94.33	77.48	**85.08**
	SVIM	91.90	94.16	93.01	91.73	94.59	**93.14**
	SVision	93.31	83.95	88.33	93.20	86.25	**89.58**
PacBio HiFi	cuteSV	97.20	94.61	95.89	97.18	95.56	**96.36**
	PBSV	86.46	82.20	84.28	86.33	93.25	**89.66**
	Sniffles2	97.85	92.50	95.10	97.78	92.97	**95.31**
	SVIM	96.89	91.67	94.21	96.74	92.63	**94.64**
	SVision	96.24	90.22	93.14	96.10	91.18	**93.58**
ONT	cuteSV	97.41	94.61	95.99	97.34	95.01	**96.16**
	PBSV	88.21	85.34	86.75	87.64	87.43	**87.53**
	Sniffles2	97.17	93.70	95.40	97.10	93.93	**95.49**
	SVIM	95.98	90.55	93.20	95.86	91.30	**93.52**
	SVision	94.95	80.43	87.16	94.90	82.20	**88.09**

Precision, recall, and F1 score in SV calling. The bold in the table means the best results. The reads are from PacBio CLR, PacBio HiFi, and ONT of sample HG002.

a,b The proportion of TP numbers in the benchmark SV callset and detected SVs.

**Figure 4.**
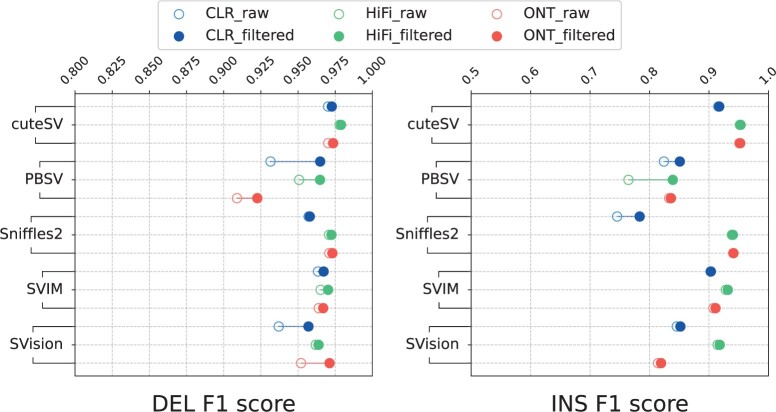
The F1 scores of different SV types before and after CSV-Filter filtering. The experiments were performed on the long read HG002 sample, including PacBio CLR, PacBio HiFi, and ONT reads. Hollow and solid points represent the F1 scores before and after filtering, respectively.

Figure 4 shows the F1 scores for different SV types before and after filtering. The figure shows that CSV-Filter performs better on INS variants. Additionally, its performance is negatively correlated with the accuracy of the dataset, meaning that it is more effective for datasets with lower accuracy (e.g., PacBio CLR). Both INS variants and low-accuracy datasets tend to have a higher number of false positives in their detection results. The experimental results indicate that CSV-Filter tends to perform better in scenarios with higher false positive rates. Detailed results of CSV-Filter’s filtering performance on different variant types in long read data can be found in Supplementary Figures S13 and S14, and Supplementary Tables S10 and S11.

We also tested CSV-Filter’s performance in the CHM13 cell line. CHM13 includes a complete end-to-end assembly, providing a high-quality human genome reference. We used Dipcall (Li *et al.* 2018) to generate an assembly-based SV call set on the CHM13 assembly and selected Dipcall’s high-confidence regions as the “ground truth”. The experiments were performed on PacBio CLR, PacBio HiFi, and ONT reads. The filtering results for different SV types are shown in Table 2, S12, and S13 as well as Supplementary Figures S15–S17. The experimental results show that the precision significantly increases, while the recall remains almost unchanged. Specifically, for PBSV, the precision for total SV types across the three alignment results increases by 9.47%, 14.11%, and 5.32%, respectively. This indicates that CSV-Filter can effectively support the T2T assemblies, and higher quality reference can further enhance the filtering performance of CSV-Filter.

**Table 2.** The filtering performance of CSV-Filter for Telomere-to-Telomere assembly of CHM13 long reads.

Datasets	Caller	Raw			Filtered		
		Recall^a^ (%)	Precision^b^ (%)	F1 score (%)	Recall (%)	Precision (%)	F1 score (%)
PacBio CLR	cuteSV	79.65	91.95	85.36	79.45	93.48	**85.81**
	PBSV	72.42	80.27	76.14	72.23	89.74	**80.08**
	Sniffles2	68.61	89.46	77.66	68.55	95.47	**79.93**
PacBio HiFi	cuteSV	82.67	91.78	86.99	82.66	92.07	**87.14**
	PBSV	69.06	78.31	73.39	69.03	92.42	**79.01**
	Sniffles2	66.44	84.31	74.31	66.43	94.84	**78.13**
ONT	cuteSV	83.98	90.09	86.93	83.89	91.71	**87.65**
	PBSV	73.52	81.21	77.17	72.43	86.53	**78.85**
	Sniffles2	70.18	81.55	75.44	70.02	91.52	**79.34**

Recall, precision, and F1 score in SV calling. The bold in the table means the best results. The reads are from PacBio CLR, PacBio HiFi, and ONT of T2T-CHM13. SV callsets were benchmarked in the high-confidence regions suggested by Dipcall (Li *et al*., 2018).

a,b The proportion of TP numbers in the benchmark SV callset and detected SVs.

The above results indicate that CSV-Filter has good generalizability and can filter detection results called from various long reads. Additionally, the filtering effect is more pronounced when the number of false positives in the detection results is high.

### 3.4 Filtering performance for short reads

We further evaluated the filtering performance of CSV-Filter for short reads. We compared CSV-Filter with DeepSVFilter, a deep learning-based SV filtering tool for short reads. In the experiments, we used short read SV detection tools including DELLY (Rausch *et al.* 2012) (v1.1.5), LUMPY (Layer *et al.* 2014) (v0.2.13), Manta (Chen *et al.* 2016) (v1.6.0), SvABA (Wala *et al.* 2018) (v1.2.0), and Cue (Popic *et al.* 2023) (v0.7.0). As the DeepSVFilter paper did not provide specific tool-based metrics for TP, FP, and other results, we also used Truvari for comparative analysis to ensure result uniformity.

Table 3 shows the filtering performance of CSV-Filter and DeepSVFilter for deletion variants in short reads. For the detection results of DELLY, CSV-Filter improved the precision by 14.65% while keeping the recall almost unchanged. For the detection results of LUMPY, Manta, SvABA, and Cue, DeepSVFilter’s precision is higher than that of CSV-Filter, but its recall significantly decreases, indicating that DeepSVFilter loses some true positives while filtering out false positives. Conversely, CSV-Filter’s recall remains almost unchanged, indicating a better filtering effect. The F1 scores further support this analysis. The changes in the number of SVs before and after filtering could refer to Supplementary Table S14.

**Table 3.** The filtering performance of CSV-Filter for HG002 short reads.

SV caller	Without filtering			SV filter	With filtering		
	Recall^a^ (%)	Precision^b^ (%)	F1 score (%)		Recall (%)	Precision (%)	F1 score (%)
DELLY	32.29	77.23	45.46	CSV-Filter	31.53	91.88	**46.95**
				DeepSVFilter	26.34	73.15	38.73
LUMPY	57.84	81.14	67.51	CSV-Filter	57.73	82.10	**67.86**
				DeepSVFilter	43.32	84.25	57.22
Manta	72.20	93.60	81.47	CSV-Filter	71.80	94.04	**81.43**
				DeepSVFilter	63.73	95.64	76.49
SvABA	34.00	64.80	44.72	CSV-Filter	33.51	89.30	**48.71**
				DeepSVFilter	31.27	98.13	47.43
Cue^c^	92.54	96.88	94.66	CSV-Filter	92.54	97.64	**95.02**
				DeepSVFilter	64.93	98.86	78.39

The bold in the table means the best results. The reads are from Illumina of sample HG002.

a,b The proportion of TP numbers in the benchmark SV callset and detected SVs.

c Cue is designed for detecting long SVs (Popic *et al*. 2023), and the results in the table are for the SVs longer than 5,000 bp.

The results indicate that CSV-Filter’s image encoding retains more SV information compared to DeepSVFilter. Meanwhile, the models generated by CSV-Filter exhibit a better capacity to learn the mapping relationship from features to SVs.

## 4. Conclusion

In this article, we proposed a novel deep learning-based SV filtering method, CSV-Filter. CSV-Filter encodes the CIGAR strings into images and adopts fine-tuning with a self-supervised model for model training. Experiments on real datasets show that CSV-Filter has good discriminative performance and can significantly reduce false positive SV calls. It also exposes strong generalization capabilities, which could filter results for both short reads and long reads.

Although there are a lot of publicly available SV call sets, big and balanced datasets suitable for training are still very limited. Moreover, these datasets usually only contain INS and DEL types of variants. To address this issue, we can construct high-confidence simulated datasets to compensate for the lack of labeled real data. Additionally, the quality of alignment results could affect the filtering performance, because the alignment accuracy may decrease for repetitive sequences, highly polymorphic regions, or complex genomic structures, thereby affecting subsequent detection and filtering. We will consider refining alignments in these complex regions.

CSV-Filter can also support sequencing data of other species. In future work, we will train new models for different species to further enhance the generality of the models.

## Supplementary Material

btae539_Supplementary_Data
