# Supplementary material for: CSV-Filter: a deep learning-based comprehensive structural variant filtering method for both short and long reads
Source: Bioinformatics. 2024 Sep 6;40(9):btae539. doi: 10.1093/bioinformatics/btae539 (PMC11419953; doi:10.1093/bioinformatics/btae539)
Supplement: btae539_Supplementary_Data [file btae539_supplementary_data.pdf]

CSV-Filter: a deep learning-based comprehensive structural variant filtering method for both short and long reads: supplementary document

This paper proposes a deep learning-based structural variant filtering tool. In this tool, we encoded CIGAR information from sequence alignment files into images and employed deep learning to perform classification of structural variants.

CONTENTS

1 Summary **2**
- A Datasets . . . 2
- B Models . . . 2
  - B.1 CNN . . . 2
  - B.2 Self-supervised models . . . 3
- C Supplementary materials . . . 3
  - C.1 Supplementary algorithms . . . 3
  - C.2 Supplementary figures . . . 3
  - C.3 Supplementary tables . . . 4
  - C.4 Supplementary notes . . . 5

2 CSV-Filter installation and usage **6**
- A Requrements . . . 6
- B Installation . . . 6
- C Usage . . . 6

3 Data availability **8**
- A HG002 . . . 8
- B NA12878 . . . 9
- C T2T-CHM13 . . . 9
- D The Ashkenazim Trio . . . 9

4 Metrics **10**

5 Supplementary algorithm **11**

6 Supplementary figures **12**

7 Supplementary tables **24**

8 Supplementary notes **37**
- A Software versions . . . 37
- B Execution parameters . . . 37
  - B.1 Aligner . . . 37
  - B.2 SV detect/filter tools . . . 38
  - B.3 Evaluation tool . . . 41

1. SUMMARY

A. Datasets

To comprehensively evaluate the performance of CSV-Filter, we used the sample HG002 [1], the sample NA12878 [2], and the T2T-CHM13 in our experiments. The sample HG002 was used for model training and filter performance evaluation, the sample NA12878 served as a supplementary evaluation of filter performance, and the T2T-CHM13 was used to assess whether CSV-Filter supports the recent datasets and to evaluate its performance.

The reasons for using the sample HG002 as the model training and evaluation standard are mainly as follows:

- HG002 provides a very robust benchmark, which can better train the model.
- HG002 has been widely used in many caller benchmarks, providing high credibility.
- HG002 offers corresponding alignment sequences for PacBio CLR, PacBio HiFi, and ONT, which can be used to compare the impact of different alignment sequences on the results.

Sample NA12878 is widely employed in genomics research and bioinformatics evaluations. The reasons for selecting this sample as supplementary evaluation are as follows:

- This sample includes a high confidence structural variant VCF file, which can be utilized to evaluate the model accuracy and filtering performance of the trained model.
- The sample was generated by the third-generation sequencing platform PacBio, allowing for the validation of the tool's performance with respect to third-generation sequencing results.
- The sample is based on the hg19 reference genome, containing a wealth of biological information, ensuring consistency with previous research.

We also employed the NA12878-Illumina [3] to compare the filtering performance between CSV-Filter and DeepSVFilter as supplementary evaluation. The reasons for selecting this sample are as follows:

- Using a dataset consistent with that employed in DeepSVFilter's filtering allows us to control experimental variables, facilitating a more robust comparison of the filter performance between CSV-Filter and DeepSVFilter.
- This paper introduces CSV-Filter as a comprehensive structural variant filtering tool suitable for both second-generation and third-generation sequencing data. The dataset utilized here was generated using the second-generation Illumina platform HiSeq, enabling us to indirectly demonstrate the applicability of CSV-Filter across different datasets and sequencing platforms.

The main reasons of using the T2T-CHM13 for testing include the following three aspects:

- T2T assemblies strive for completeness, aiming to cover all regions of the genome from one telomere (end) to the other.
- T2T assemblies are designed to be highly accurate, minimizing errors in sequence and structural organization.
- Some false positives turned out to be true variants that are supported by recent T2T assemblies

For detailed descriptions of these samples see section 3.

B. Models

B.1. CNN

MobileNet v2 and ResNet models are based on the PyTorch framework and are pre-trained using the ImageNet dataset. The ImageNet dataset is a large-scale visual dataset containing over 14 million annotated images spanning 1000 categories. This extensive dataset enables the models to learn rich feature representations, ensuring outstanding performance in various visual tasks [4].

Pretraining on ImageNet allows the models to learn both low-level and high-level image features, reducing the need for training from scratch and improving performance. In many computer vision applications, such as image classification, object detection, semantic segmentation, and image generation, using models pre-trained on ImageNet has become standard practice.

MobileNet v2 is a lightweight CNN designed with core features such as depthwise separable convolutions, inverted residual structures, and linear bottlenecks with shortcut connections. These core designs reduce the model's parameter count while maintaining high accuracy, making it suitable for mobile and embedded devices.

ResNet (Residual Network) is a deep convolutional neural network known for its introduction of residual modules, which significantly enhance performance and stability. ResNet34 and ResNet50 are two versions of ResNet, differing in depth, with 34 layers and 50 layers, respectively.

ResNet34 is composed of fewer convolutional layers, including 3 convolutional layers and 4 residual block groups, each containing 3, 4, 6, and 3 residual units. ResNet34 efficiently extracts shallow features, making it suitable for moderately complex image classification tasks.

ResNet50 is composed of more convolutional layers, including 1 convolutional layer and 4 residual block groups, each containing 3, 4, 6, and 3 residual units. ResNet50 extracts more advanced and richer features, capturing more image details.

CSV-Filter uses a multi-level grayscale image encoding based on CIGAR string, mapping alignment information into two-dimensional images. Since SVs represent abnormal segments in sequences, images generated in SV regions will have different characteristics compared to those in normal regions. Models pre-trained on ImageNet already possess strong feature learning capabilities. Although the visual features of these images differ from natural images, some universal features still exist. For example, different types of SVs form specific patterns and structures in images, which can be detected by the high-level features in pre-trained models. With the feature learning capabilities from ImageNet, these models can distinguish between images containing SVs and normal images, thus achieving SV classification.

B.2. Self-supervised models

The three self-supervised models were trained based on the ResNet50 model, with the output representations of the models regularized using VICReg. We tested the original-sized ResNet50 model, ResNet50(x2) model with doubled width, and ResNet200(x2) model with increased parameters.

We used VICReg for model training in CSV-Filter to prevent collapse during training while maintaining information content and achieving good generalization. VICReg addresses the collapse issue through three regularization terms: variance, covariance, and invariance:

- Variance regularization maintains the variance of each embedding dimension above a certain threshold, preventing all inputs from mapping to the same vector.
- Covariance regularization reduces the covariance between pairs of embedding variables to near 0, decorrelating the variables and preventing information redundancy.
- Invariance regularization: minimizes the distance between the embedding vectors of different views of the same image.

C. Supplementary materials

C.1. Supplementary algorithms

Supplementary algorithms provide a more detailed process of relevant algorithm within the original text.

- Algorithm S1 shows the generation of negative samples.
- Algorithm S2 shows the process of image encoding.

Train.

C.2. Supplementary figures

Supplementary figures provide a more detailed exposition of relevant content within the original text.

- Fig. S1 describes the operation of the classification layer, encompassing attentional fully connected units, fully connected units, and the fully connected classification units.

- Fig. S2 counts the distribution of CIGAR strings in NA12878-PacBio and presents the proportional representation of each CIGAR type in the form of pie charts.
- Fig. S3 shows the image space occupancy of CSV-Filter with multi-channel and multi-level grayscale image encoding in sample NA12878.
- Fig. S4 compares the training time for 50 epochs of CSV-Filter with and without mixed precision in CNN models MobileNet_v2, ResNet34, and ResNet50.
- Fig. S5 compares the GPU memory used of CSV-Filter with and without mixed precision in CNN models MobileNet_v2, ResNet34, and ResNet50.
- Fig. S6 $\sim$ S8 show the training regression of CNN models MobileNet_v2, ResNet34, and ResNet50, respectively.
- Fig. S9 $\sim$ S11 show the training regression of self-supervised models ResNet50, ResNet50(x2), and ResNet200(x2), respectively.
- Fig. S12 shows the Tier1 benchmark SV callset and high confidence HG002 region SV length distribution.
- Fig. S13 and S14 show the Recall and Precision before and aftering filtering for callers in sample HG002.
- Fig. S15 $\sim$ S17 show the Recall, Precision, and F1 score before and aftering filtering for callers in T2T-CHM13, respectively.

C.3. Supplementary tables

Supplementary tables provide additional elaboration on the experimental data presented in the original text.

- Table S1 shows the proportion of CIGAR operations in the alignment files of NA12878-PacBio, NA12878-Illumina, HG002, HG003, and HG004 samples.
- Table S2 offers supplementary details on the parameters of the one-dimensional attentional residual module within the classification layer.
- Table S3 offers the specific configuration of the server.
- Table S4 offers the detailed information about the 9 used models.
- Table S5 shows the Accuracy, micro-average values of precision, recall, and F1 score, as well as F1 score for INS, DEL, and NEG of CSV-Filter with different models on sample HG002.
- Table S6 shows the different classification result metrics of CSV-Filter in CNN models MobileNet_v2, ResNet34, and ResNet50 for sample HG002, respectively.
- Table S7 shows the different classification result metrics of CSV-Filter in self-supervised models ResNet50, ResNet50(x2), and ResNet200(x2) for sample HG002, respectively.
- Table S8 shows the different classification result metrics of CSV-Filter in CNN models MobileNet_v2, ResNet34, and ResNet50 for sample NA12878, respectively.
- Table S9 shows the different classification result metrics of CSV-Filter in self-supervised models ResNet50, ResNet50(x2), and ResNet200(x2) for sample NA12878, respectively.
- Table S10 and S11 show the filtering performance of CSV-Filter on deletion and insertion variants for sample HG002 long reads.
- Table S12 and S13 show the filtering performance of CSV-Filter on deletion and insertion variants for T2T-CHM13.
- Table S14 compares the filtering performance of DeepSVFilter and CSV-Filter on deletion variants for sample HG002 short reads.

- Table S15 compares the Accuracy, micro-average values of Precision, Recall, and F1 score, as well as F1 score for NEG, DEL, and INS of CSV-Filter with and without mixed precision on different models on sample HG002.
- Table S16 compares Accuracy, micro-average values of Precision, Recall, and F1 score, as well as F1 score for NEG, DEL, and INS of CSV-Filter with multi-channel image and grayscale image on different models on sample NA12878.
- Table S17 shows the filter performance CSV-Filter on the SV results generated by structural variant callers PBSV and Sniffles2 with trained self-Supervised models ResNet50, ResNet50(x2), and ResNet200(x2) on sample NA12878.

C.4. Supplementary notes

Supplementary notes provide descriptions of the different versions of tools and the detailed execution commands used during comparative testing experiments.

2. CSV-FILTER INSTALLATION AND USAGE

A. Requrements

CSV-Filter is tested to work under:

- Ubuntu 18.04
- gcc 7.5.0
- CUDA 11.3
- Anoconda 4.10.3
- Python 3.6
- pysam 0.15.4
- pytorch 1.10.2
- pytorch-lightning 1.5.10
- hyperopt 0.2.7
- matplotlib 3.3.4
- numpy 1.19.2
- pudb 2022.1.3
- redis 4.3.6
- samtools 1.5
- scikit-learn 0.24.2
- torchvision 1.10.2
- tensroboard 2.11.2

B. Installation

For a detailed installation process, please refer to the CSV-Filter's GitHub documentation at https://github.com/xzyschumacher/CSV-Filter

C. Usage

After installation, CSV-Filter can be run with the following command:

Simple train.

```
$ python simple_train.py ${selected_model}
```

Train.

(1) VCF data preprocess:
```
$ python vcf_data_process.py
```

(2) BAM data preprocess:
```
$ python bam2depth.py
```

(3) Parallel generate images:
```
$ python parallel_process_file.py -thread_num ${thread_num}
```

(4) Check generated images:
```
$ python process_file_check.py
```

(5) Rearrange generated images:
```
$ python data_spread.py
```

(6) Train:
```
$ python train.py
```

Predict & Filter.

(1) Predict:
```
$ python predict.py ${selected_model}
```

(2) Filter:
```
$ python filter.py ${selected_model}
```

3. DATA AVAILABILITY

In this study, we utilized the sample HG002 and NA12878 from NIST's Genome in a Bottle (GIAB) project to train the CSV-Filter model. We also tested the filter performance of CSV-Filter on recent T2T-CHM13. The reference sequences, alignment files, and benchmark SV callset for the samples are provided below:

A. HG002

The benchmark SV callset of sample HG002, HG002_SVs_Tier1_v0.6, comes from the paper "A robust benchmark for detection of germline large deletions and insertions." This benchmark SV callset integrates variant calls from 19 sequence-resolved methods across different technologies. A new method named SVanalyzer clusters candidate SVs, grouping SVs with estimated similar sequence changes. SVs are then filtered to retain those supported by multiple technologies and by five or more callsets from a single technology (e.g., Bionano or Nabsys). Genotyping and further filtering were conducted to define the benchmark regions [1]. The Tier 1 benchmark SV callset covers 2.51 Gbp and includes 4,199 deletions and 5,442 insertions. This benchmark SV callset has been widely used in SV detection tools like Sniffles2, SVision, and DeBreak.

- Tier1 benchmark SV callset and high-confidence HG002 region can be freely downloaded from: https://ftp-trace.ncbi.nlm.nih.gov/ReferenceSamples/giab/data/AshkenazimTrio/analysis/NIST_SVs_Integration_v0.6/

In this study, third-generation data used include PacBio 70x (CLR), PacBio CCS 15kb_20kb chemistry2 (HiFi), and Oxford Nanopore ultralong (guppy-V3.2.4_2020-01-22) datasets. The average read length for PacBio CLR is 5,894.1 bp with a sequencing depth of 65.4349. PacBio HiFi has an average read length of 11,562.6 bp with a sequencing depth of 56.023. ONT data have an average read length of 11,043.5 bp with a sequencing depth of 56.1009. These datasets have been used in various SV detection tools such as Sniffles2, SVision, and DeBreak, as well as in review papers, ensuring high reliability.

- PacBio 70x (CLR) file can be freely downloaded from: https://ftp-trace.ncbi.nlm.nih.gov/ReferenceSamples/giab/data/AshkenazimTrio/HG002_NA24385_son/PacBio_MtSinai_NIST/

- PacBio CCS 15kb_20kb chemistry2 (HiFi) file can be freely downloaded from: https://ftp-trace.ncbi.nlm.nih.gov/ReferenceSamples/giab/data/AshkenazimTrio/HG002_NA24385_son/PacBio_CCS_15kb_20kb_chemistry2/reads/

- Oxford Nanopore ultralong (guppy-V3.2.4_2020-01-22) can be freely downloaded from: ftp://ftp-trace.ncbi.nlm.nih.gov/ReferenceSamples/giab/data/AshkenazimTrio/HG002_NA24385_son/Ultralong_OxfordNanopore/guppy-V3.2.4_2020-01-22/HG002_ONT-UL_GIAB_20200122.fastq.gz

The second-generation data we used is from Illumina. This dataset has been used in papers like Cue and LinkedSV, ensuring high reliability. The average read length for Illumina is 148 bp with a sequencing depth of 63.2589.

- Illumina data can be freely downloaded from: https://ftp-trace.ncbi.nlm.nih.gov/giab/ftp/data/AshkenazimTrio/HG002_NA24385_son/NIST_HiSeq_HG002_Homogeneity-10953946/NHGRI_Illumina300X_AJtrio_novoalign_bams/HG002.hs37d5.60x.1.bam

Third-generation sequences were aligned using minimap2, pbmm2, and NGMLR to the reference sequence GRCh37. Second-generation sequences were aligned using BWA-MEM to the reference sequence hs37d5.

- Reference genome GRCh37 can be freely downloaded from: https://ftp.ensembl.org/pub/release-75/fasta/homo_sapiens/dna/Homo_sapiens.GRCh37.75.dna.primary_assembly.fa.gz

- Reference genome hs37d5 can be freely downloaded from: https://ftp-trace.ncbi.nih.gov/1000genomes/ftp/technical/reference/phase2_reference_assembly_sequence/hs37d5.fa.gz

B. NA12878

NA12878-PacBio. provided by NCBI are available through the Genome in a Bottle Consortium website. It is sequenced by the third-generation platform PacBio was sequenced to 44x coverage on a third-generation platform, PacBio. This sample has an average sequencing length of 5096 bp. The reads were aligned to the hg19 human reference genome using blasr v1.3.2. The sample includes a VCF file containing SVs produced using three different variant detection methods: PBHoney [5], a custom pipeline [6], and methodology from [7] for assembled sequences. The SVs detected in the VCF file includes INS or DEL.

- Reference genome hg19 can be freely downloaded from: http://hgdownload.cse.ucsc.edu/goldenPath/hg19/bigZips/chromFa.tar.gz
- The alignment file can be freely downloaded from: https://ftp.ncbi.nlm.nih.gov/giab/ftp/data/NA12878/NA12878_PacBio_MtSinai/sorted_final_merged.bam
- The high confidence structural variants can be freely downloaded from: https://ftp.ncbi.nlm.nih.gov/giab/ftp/data/NA12878/NA12878_PacBio_MtSinai/NA12878.sorted.vcf.gz

NA12878-Illumina. is provided by Illumina Platinum Genomes Project [8]. It is sequenced by the second-generation platform Illumina HiSeq was sequenced to 50x coverage on a next-generation platform, Illumina HiSeq. This sample has an average sequencing length of 101 bp. The reads were aligned to the GRCh38DH reference genome using BWA-MEM. The alignment file can be downloaded from the European Nucleotide Archive (ENA accession: PRJEB3381).

- Reference genome GRCh38DH can be freely downloaded from: https://ftp-trace.ncbi.nih.gov/1000genomes/ftp/technical/reference/GRCh38_reference_genome/GRCh38_full_analysis_set_plus_decoy_hla.fa
- The alignment file website: https://www.ebi.ac.uk/ena/browser/view/PRJEB3381?show=reads Bam file download: ftp://ftp.sra.ebi.ac.uk/vol1/run/ERR194/ERR194147/NA12878_S1.bam
- The high confidence structural variants can be freely downloaded from: ftp://ftp.1000genomes.ebi.ac.uk/vol1/ftp/phase3/integrated_sv_map/supporting/GRCh38_positions/ALL.wgs.mergedSV.v8.20130502.svs.genotypes.GRCh38.vcf.gz.

C. T2T-CHM13

The Telomere-to-Telomere (T2T) assembly of CHM13 refers to a highly complete and contiguous genome assembly generated from the CHM13 cell line. T2T assemblies are designed to be highly accurate, minimizing errors in sequence and structural organization.

Since the T2T-CHM13 reference sequence does not have a corresponding gold standard dataset, we used the method mentioned in DeBreak's paper. We applied Dipcall (v0.3) [9] to the end-to-end assembly of CHM13 to generate an assembly-based call set. We considered SVs larger than 50bp as the ground truth call set and chose Dipcall's high-confidence regions as the "ground truth."

- T2T assembly and sequencing reads of CHM13 can be freely downloaded from: https://github.com/marbl/CHM13
- DeBreak provides the benchmark truth call set used in the paper: https://zenodo.org/records/7214225
- High-confidence regions were generated using Dipcall, instructions can be found in section B.2.

D. The Ashkenazim Trio

The Ashkenazim Trio (including HG002, HG003, and HG004) [10] are also used for counting CIGAR operations proportion. They are from SRA: SRX847862 to SRX848317, the raw sequence data, alignments, and corresponding ground truth sets for evaluation can be freely downloaded from: https://ftp.ncbi.nih.gov/giab/ftp/data/AshkenazimTrio/

4. METRICS

In this study, we chose a range of quality metrics in deep learning to evaluate the performance of the model. These metrics include the Receiver Operating Characteristic (ROC), accuracy, precision, recall, F1 score, etc.

The ROC curve is a common metric used to evaluate the classification performance of binary classification models. It illustrates the relationship between the true positive rate (TPR) and the false positive rate (FPR) of the classifier at different classification thresholds. The TPR, also known as sensitivity or recall, was calculated by Equation S1. And the FPR was calculated by Equation S2.

$$TPR = \frac{TP}{TP + FN} \tag{S1}$$

$$FPR = \frac{FP}{FP + TN} \tag{S2}$$

where TP, FN, FP, and TN denote true positives, false negatives, false positives, and true negatives, respectively.

The shape of the ROC curve and the Area Under Curve (AUC) can reflect the performance of a classifier. The AUC value ranges between 0 and 1. The closer the AUC value is to 1, the better the classifier is at correctly ranking positive instances ahead of negative instances.

$$ACC = \frac{TP + TN}{TP + TN + FP + FN} \tag{S3}$$

$$P = \frac{TP}{TP + FP} \tag{S4}$$

$$F1 = 2 \times \frac{P \times R}{P + R} \tag{S5}$$

Accuracy (ACC) measures the proportion of correctly classified samples among all samples by the classifier. Precision (P) measures the proportion of true positive samples among all samples predicted as positive by the classifier. Recall (R), also known as TPR, measures the proportion of true positive samples among all actual positive samples. The F1 score is a comprehensive evaluation metric that takes into account both precision and recall to measure the overall performance of a model. The calculation for accuracy, precision, and F1 score is shown in Equation S3, S4, and S5, respectively.

5. SUPPLEMENTARY ALGORITHM

Algorithm S1. Negative samples generation

Require: $begin_{sv}, len_{chr}, \lambda_{sv}$
Ensure: $begin_{neg}, end_{neg}$

$\lambda_{neg} \Leftarrow \lambda_{sv}$
$X \sim \text{Pois}(\lambda_{neg})$
$len_{neg} \Leftarrow \text{sampling}(X)$
while $len_{neg} > len_{chr}$ **do**
    $len_{chr} \Leftarrow \text{sampling}(X)$
    $begin_{neg} \Leftarrow \text{random}(1 \sim len_{chr})$
    while $begin_{chr} \in begin_{sv}$ **do**
        $begin_{neg} \Leftarrow \text{random}(1 \sim len_{chr})$
        $end_{neg} \Leftarrow begin_{neg} + len_{neg}$
if $end_{neg} > len_{chr}$ **then**
    $end_{neg} \Leftarrow len_{chr}$

Algorithm S2. Images encoding

Require: BAM, SV_{info}
Ensure: $img[\,][\,]$

$reads, n$ = sam_file.fetch(BAM, SV_{info})
for $i \leftarrow 0$ to $n - 1$ **do**
    $b[\,]$ = append(reads.start)
    $e[\,]$ = append(reads.end)
b_{min} = min.b
for $i \leftarrow 0$ to $n - 1$ **do**
    $offset = b[i] - b_{min}$
    for $j \leftarrow 0$ to $e[i]$ **do**
        if $offset < j < e[i]$ **then**
            if $CIGAR = M, I, D, S$ **then**
                $img[i][j]$ = kernel_cigar($CIGAR$)
            else
                $img[i][j]$ = 0
        else
            $img[i][j]$ = 0
img.resize([224, 224])

6. SUPPLEMENTARY FIGURES

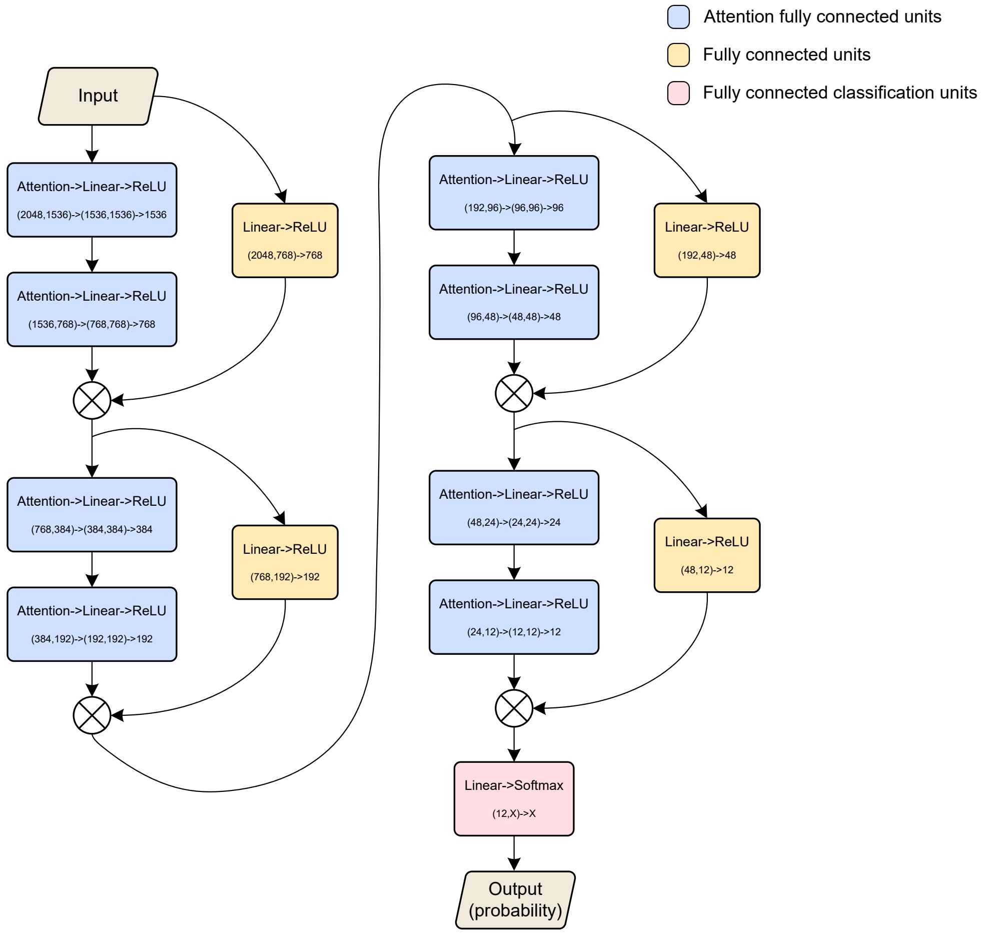

Fig. S1. Structural variant classification layer description diagram.

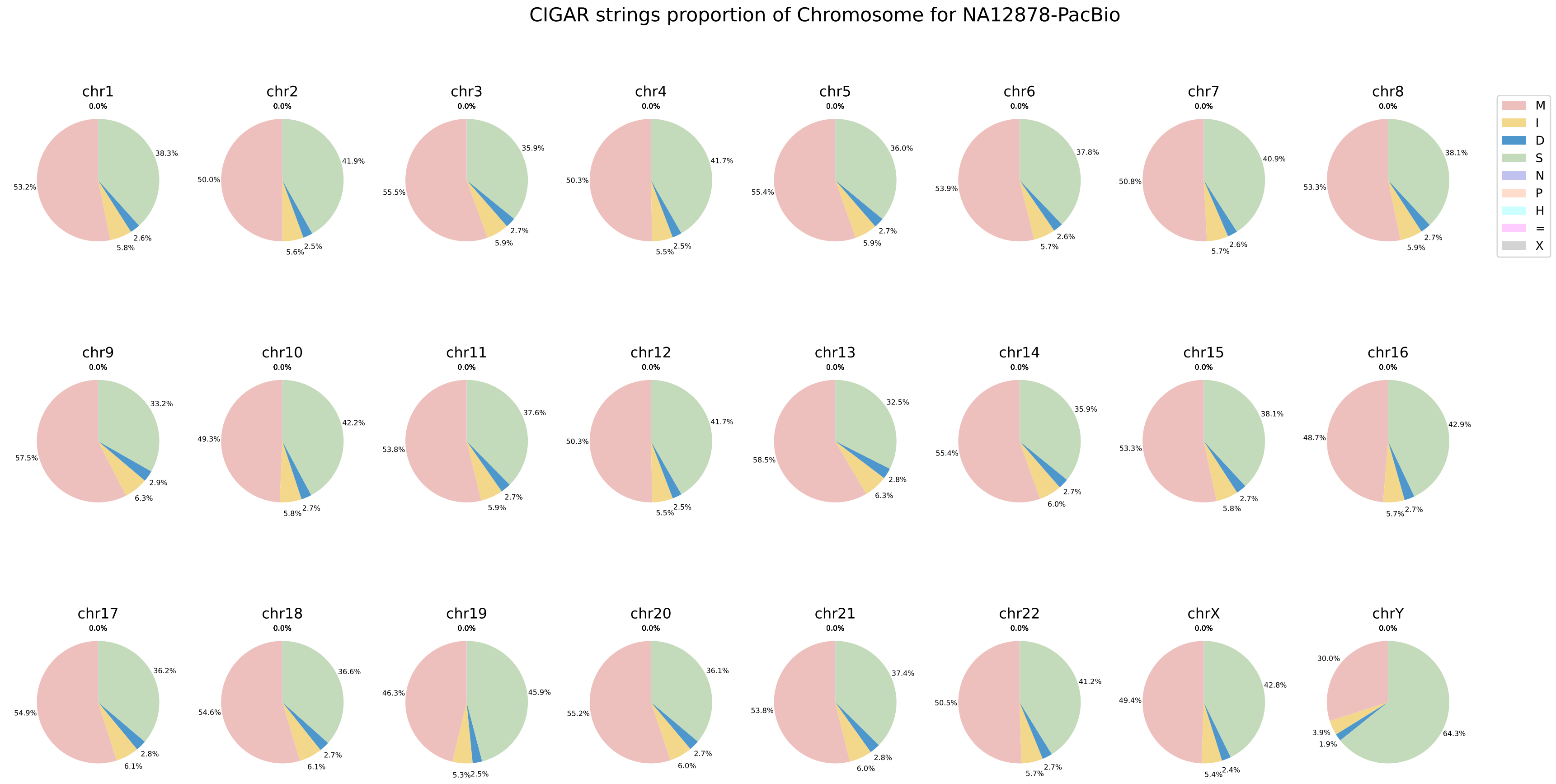

Fig. S2. The CIGAR string proportion analysis of NA12878-PacBio.

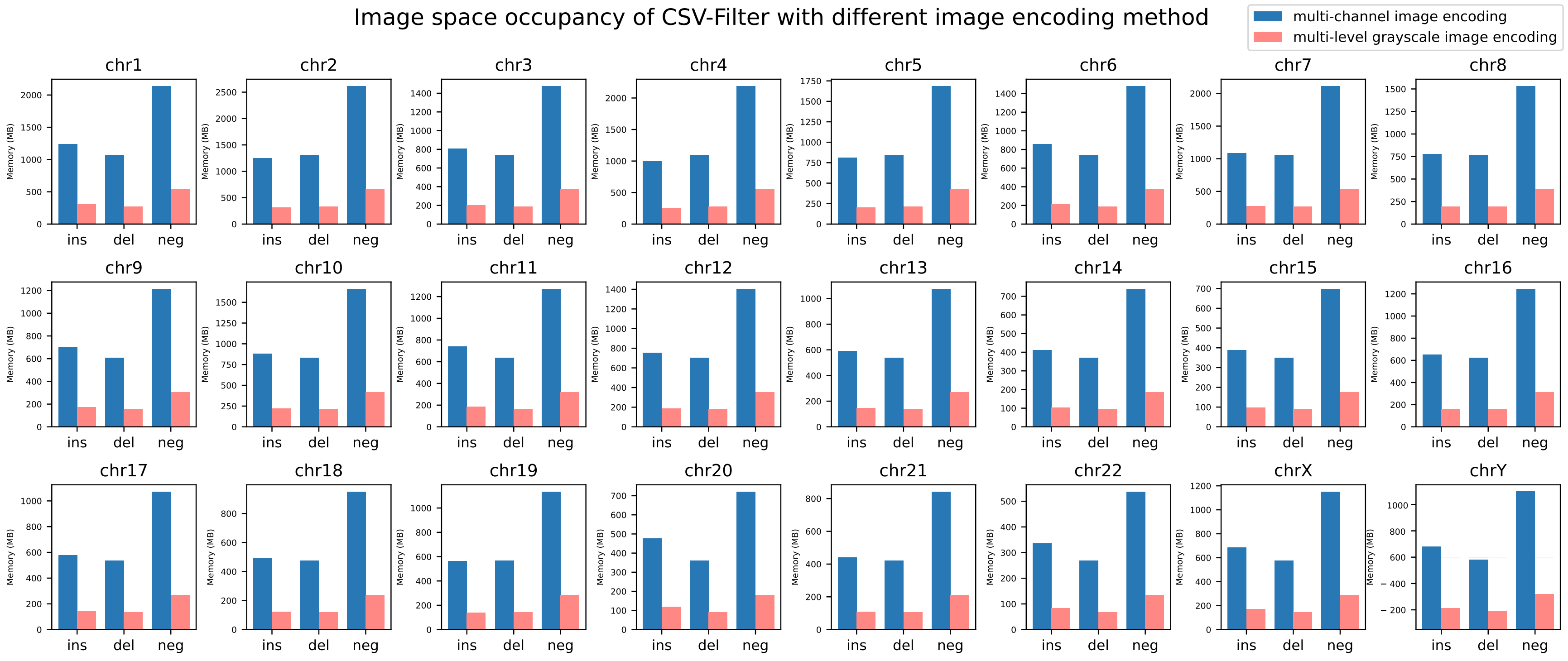

Fig. S3. Image space occupancy of CSV-Filter with multi-channel and multi-level grayscale image encoding in sample NA12878.

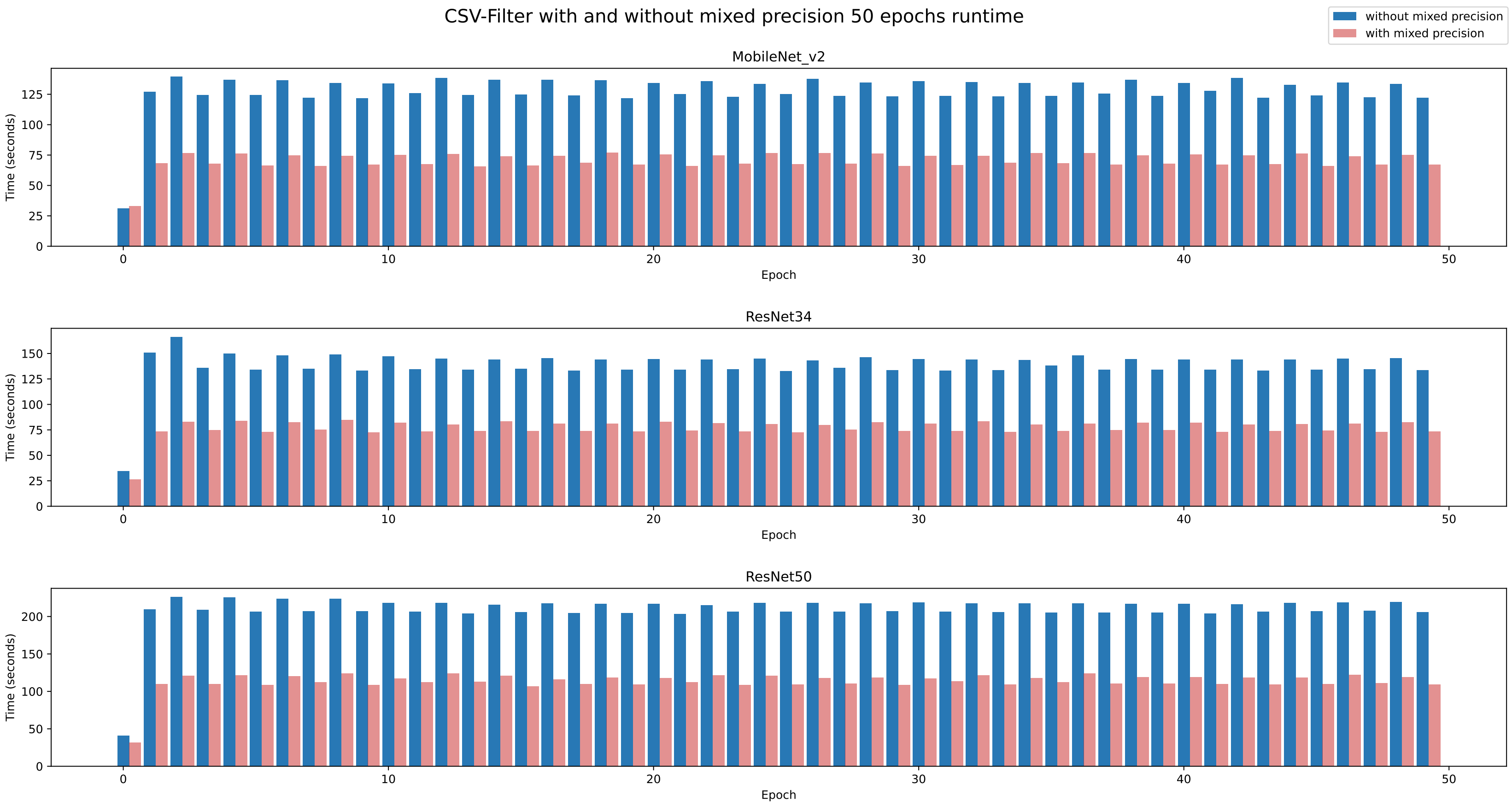

Fig. S4. Training time comparison for 50 epochs of CSV-Filter with and without mixed precision in CNN models MobileNet_v2, ResNet34, and ResNet50.

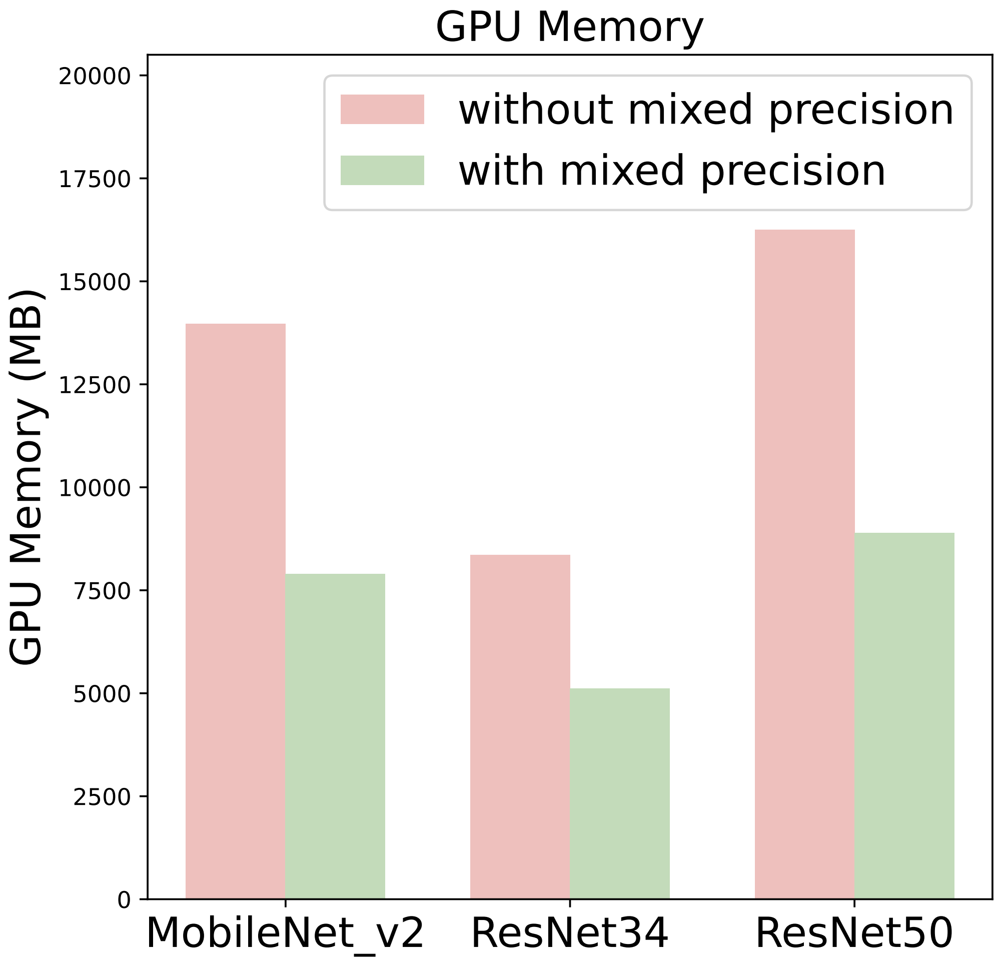

Fig. S5. GPU memory used of CSV-Filter with and without mixed precision in CNN models MobileNet_v2, ResNet34, and ResNet50.

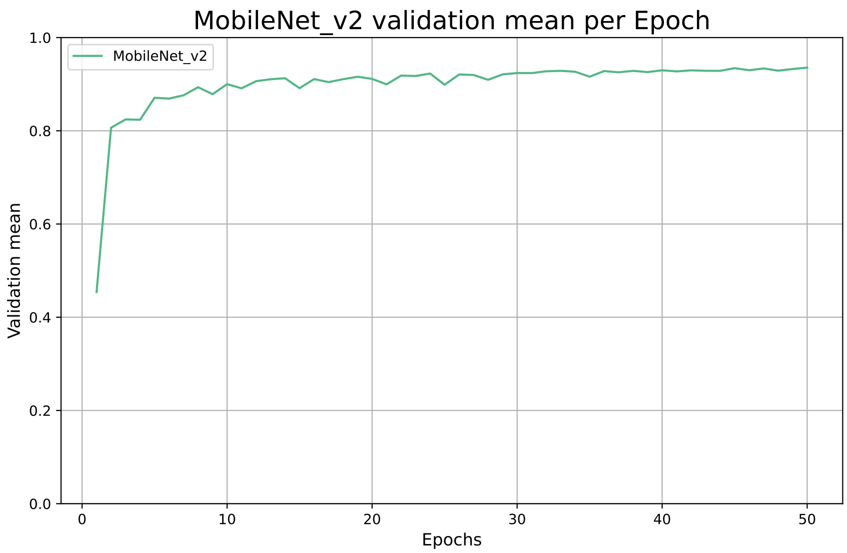

Fig. S6. CNN MobileNet_v2 model training process.

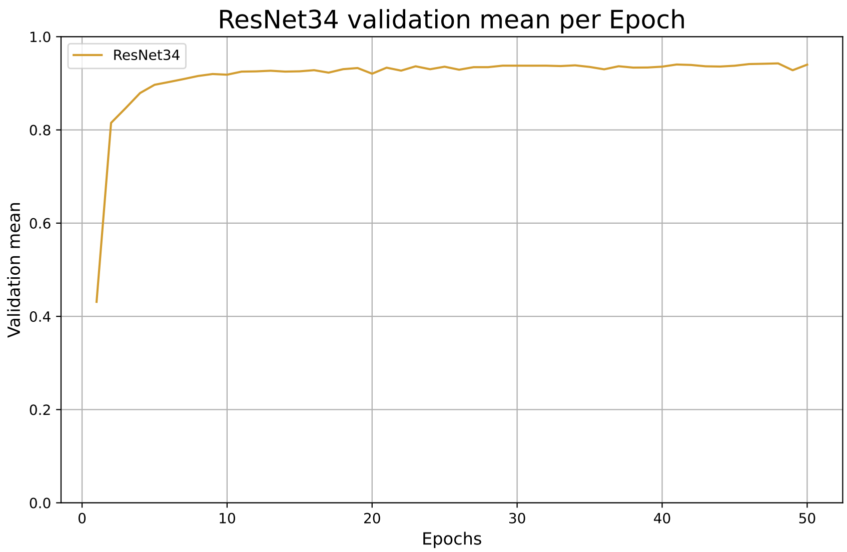

Fig. S7. CNN ResNet34 model training process.

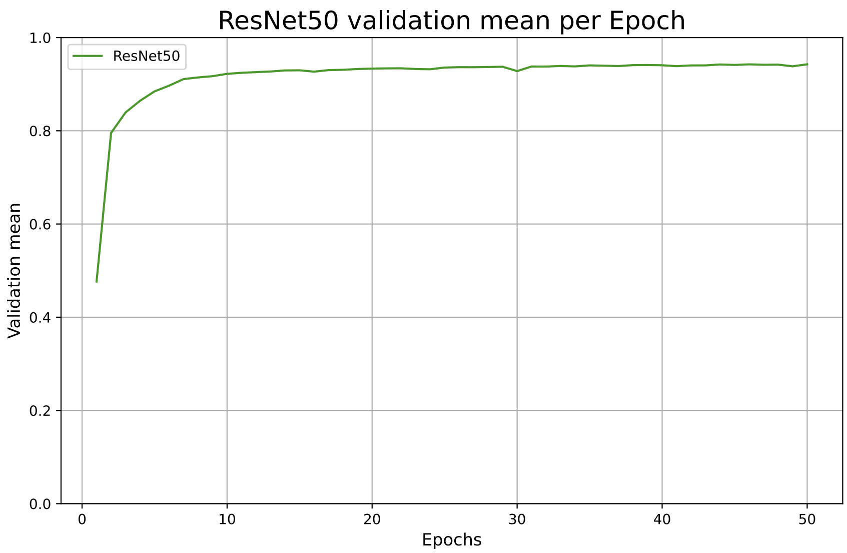

Fig. S8. CNN ResNet50 model training process.

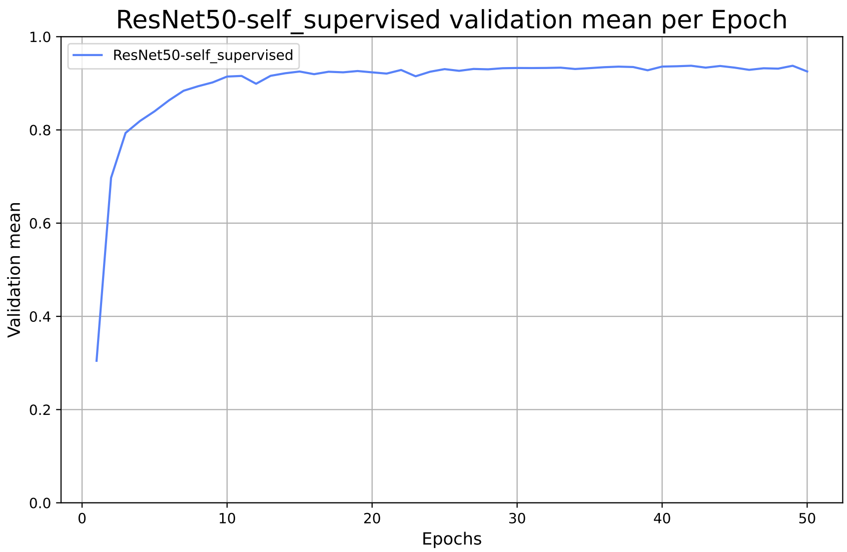

Fig. S9. Self-supervised ResNet50 model training process.

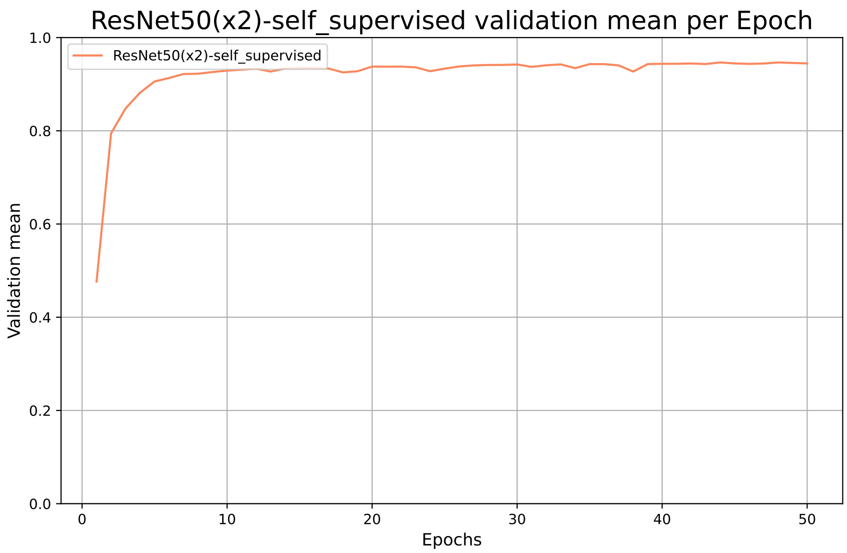

Fig. S10. Self-supervised ResNet50(x2) model training process.

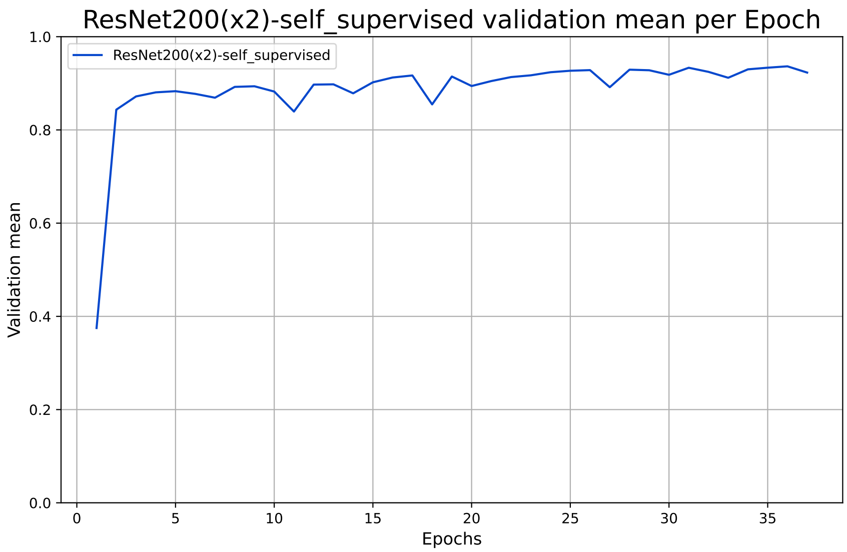

Fig. S11. Self-supervised ResNet200(x2) model training process.

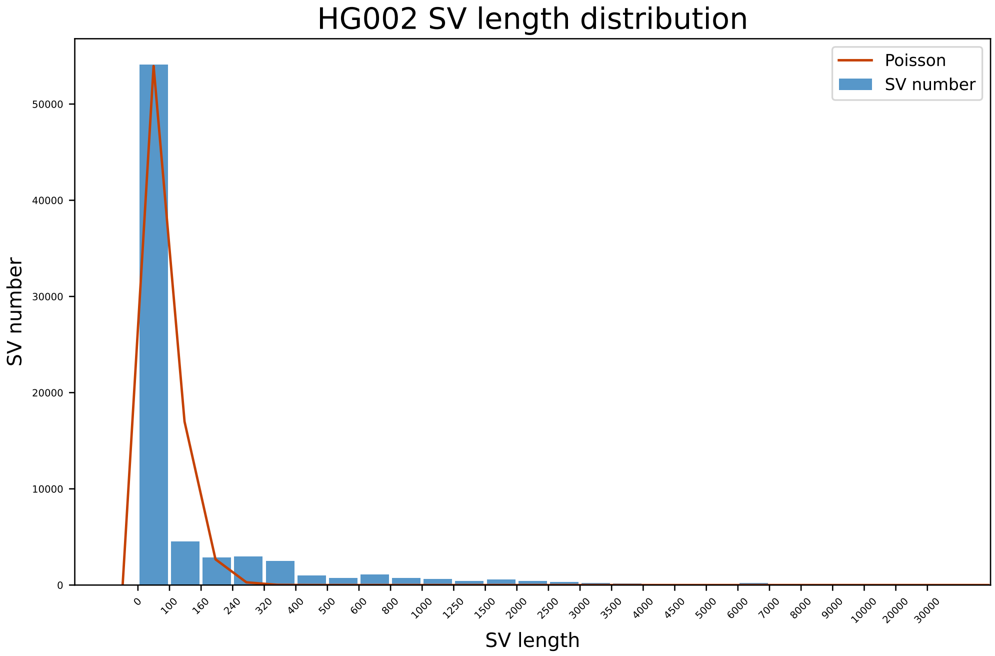

Fig. S12. The Tier1 benchmark SV callset and high confidence HG002 region SV length distribution.

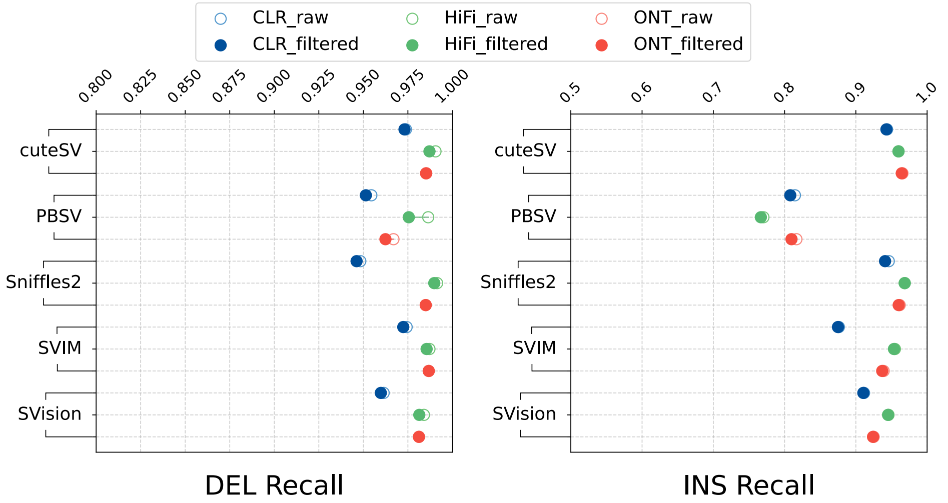

Fig. S13. The change in Recall for cuteSV, PBSV, Sniffles2, SVIM, and SVision before and after CSV-Filter filtering. The experimental sample is HG002. Hollow circles represent values before filtering, while solid circles represent values after filtering.

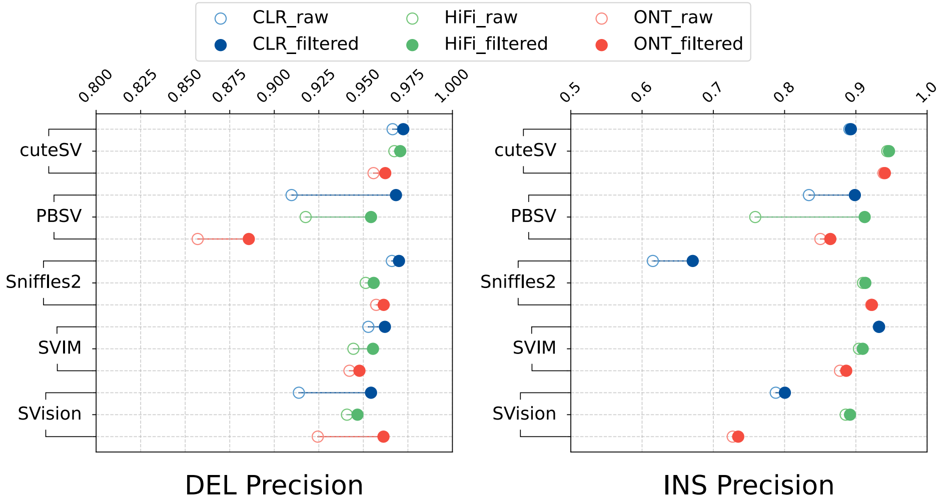

Fig. S14. The change in Precision for cuteSV, PBSV, Sniffles2, SVIM, and SVision before and after CSV-Filter filtering. The experimental sample is HG002. Hollow circles represent values before filtering, while solid circles represent values after filtering.

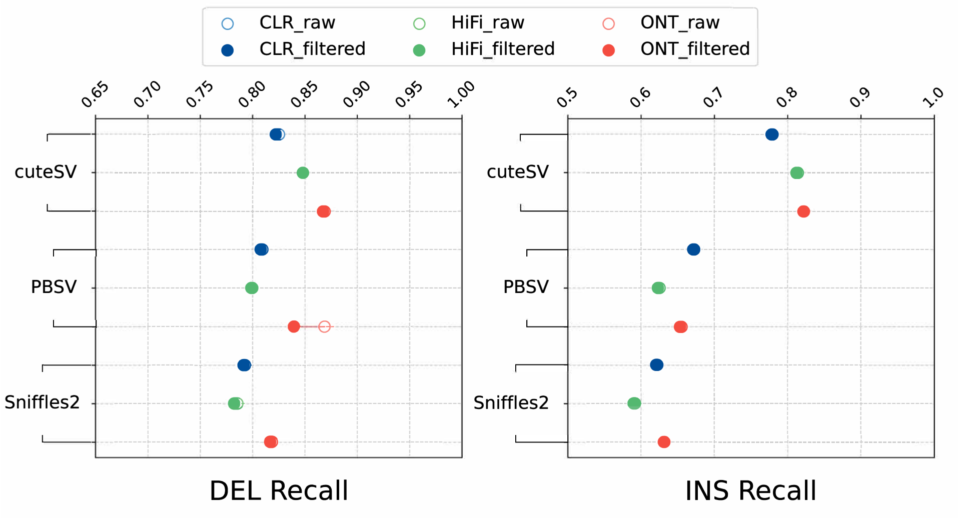

Fig. S15. The change in Recall for cuteSV, PBSV, and Sniffles2 before and after CSV-Filter filtering. The experimental sample is Telomere-to-Telomere assembly of CHM13. Hollow circles represent values before filtering, while solid circles represent values after filtering.

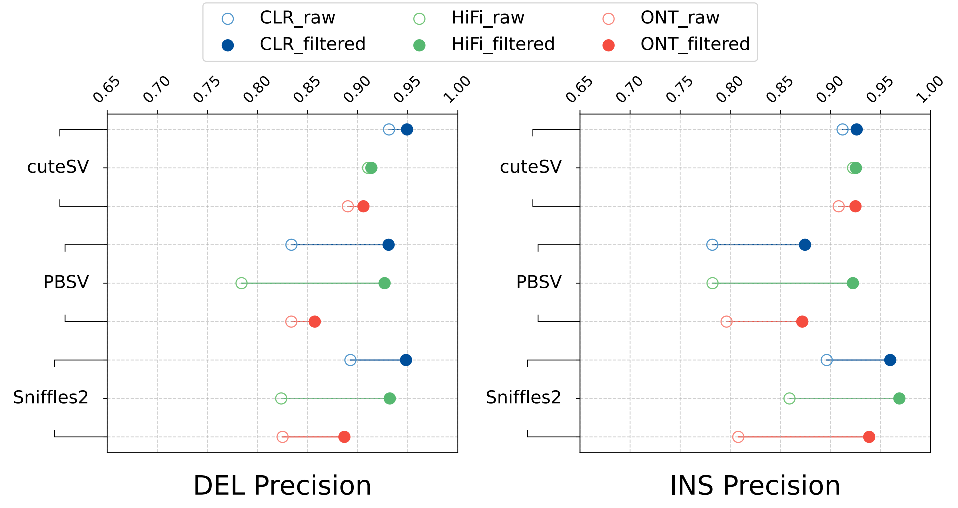

Fig. S16. The change in Precision for cuteSV, PBSV, and Sniffles2 before and after CSV-Filter filtering. The experimental sample is Telomere-to-Telomere assembly of CHM13. Hollow circles represent values before filtering, while solid circles represent values after filtering.

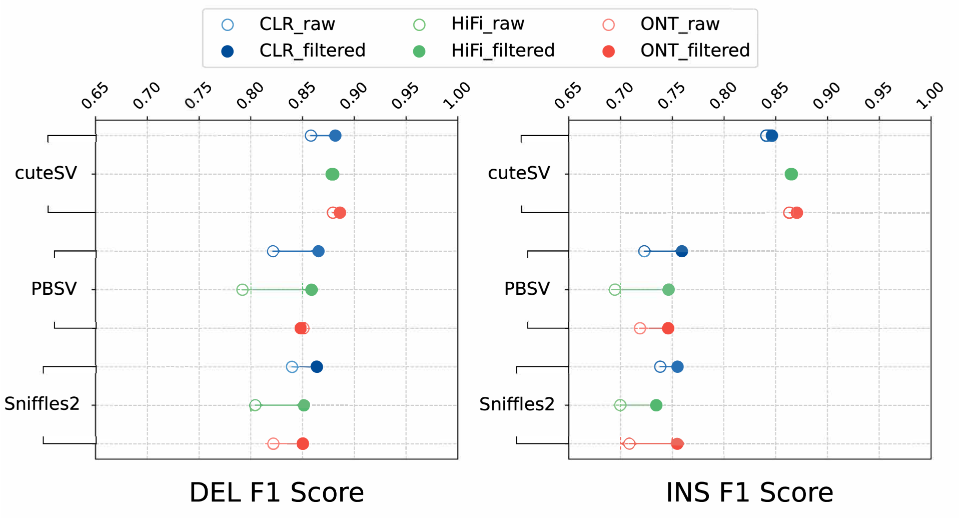

Fig. S17. The change in F1 score for cuteSV, PBSV, and Sniffles2 before and after CSV-Filter filtering. The experimental sample is Telomere-to-Telomere assembly of CHM13. Hollow circles represent values before filtering, while solid circles represent values after filtering.

7. SUPPLEMENTARY TABLES

Table S1. The CIGAR operations proportion analysis.

Sample	M	I	D	N	S	H	P	=	X
NA12878-Pacbio	52.28%	5.76%	2.64%	– –	39.33%	– –	– –	– –	– –
NA12878-Illumina	99.18%	0.02%	0.03%	– –	0.78%	– –	– –	– –	– –
PacBio HG002	53.56%	4.85%	2.08%	– –	37.93%	– –	– –	– –	1.59%
PacBio HG003	52.67%	4.90%	2.01%	– –	38.84%	– –	– –	– –	1.57%
PacBio HG004	52.05%	5.21%	1.81%	– –	39.40%	– –	– –	– –	1.53%

Table S2. One-dimensional attention residual module parameters

Name	Input dimension	Output dimension
Module 1	2048	768
Module 2	768	192
Module 3	192	48
Module 4	48	12

Table S3. Server configuration.

Items	Server_1	Server_2
CPU	Intel Core i9-12900K @ 3.2GHz	Intel Xeon Gold 6230 @ 2.10GHz
Memory	128GB	384GB
GPU	NVIDIA 3090Ti × 2	NVIDIA RTX A6000 × 2
Operating System	Ubuntu 18.04	Ubuntu 20.04
Python	3.6.13	3.6.13
PyTorch	1.10.2	1.10.2

Table S4. The details of used models.

Type	Model	Size	Param.
CNN	MobileNet v2	14.26 M	3.50×10^6
	ResNet34	87.33 M	21.80×10^6
	ResNet50	102.54 M	25.56×10^6
	ResNet50(x2)	389.96 M	102.22×10^6
	ResNet200(x2)	985.37 M	$258, 69 \times 10^6$
Self-Supervised	ResNet34	81.19 M	21.28×10^6
	ResNet50	94.34 M	23.51×10^6
	ResNet50(x2)	376.15 M	93.91×10^6
	ResNet200(x2)	1002.31 M	250.13×10^6

Table S5. Accuracy, micro-average values of precision, recall, and F1 score, as well as F1 score for INS, DEL, and NEG of CSV-Filter with different models on sample HG002.

Type	Model	Accuracy	Precision	Recall	F1 score	F1 score		
						INS	DEL	NEG
CNN	MobileNet v2	92.84%	93.89%	91.31%	92.39%	95.05%	88.95%	93.19%
	ResNet34	93.09%	93.49%	91.98%	92.63%	95.43%	89.01%	93.45%
	ResNet50	93.61%	94.60%	92.16%	93.17%	96.16%	89.57%	93.79%
	ResNet50(x2)	94.05%	94.49%	92.91%	93.62%	96.74%	89.83%	94.29%
	ResNet200(x2)	93.39%	93.97%	92.27%	92.98%	96.59%	89.10%	93.27%
Self-supervised	ResNet34	93.20%	93.61%	92.34%	92.93%	93.83%	91.19%	93.76%
	ResNet50	93.97%	94.87%	92.52%	93.52%	**96.62%**	89.75%	94.18%
	ResNet50(x2)	**94.94%**	**95.44%**	**94.12%**	**94.72%**	96.28%	**92.81%**	**95.06%**
	ResNet200(x2)	93.65%	94.44%	92.47%	93.26%	96.40%	89.78%	93.58%

The bold in the table means the best results.

Table S6. HG002 dataset. Different classification result metrics of CSV-Filter in MobileNet_v2, ResNet34, and ResNet50 CNN models.

Filter	Type	Model	Classification	Accuracy	Precision	Recall	F1 score
CSV-Filter	CNN	MobileNet v2	NEG	92.84%	89.62%	97.04%	93.19%
			DEL		95.90%	82.94%	88.95%
			INS		96.16%	93.96%	95.05%
			macro avg		93.89%	91.31%	92.39%
		ResNet34	NEG	93.09%	91.62%	95.35%	93.45%
			DEL		94.29%	84.30%	89.01%
			INS		94.57%	96.31%	95.43%
			macro avg		93.49%	91.98%	92.63%
		ResNet50	NEG	93.61%	90.59%	97.22%	93.79%
			DEL		96.73%	83.40%	89.57%
			INS		96.46%	95.85%	96.16%
			macro avg		94.60%	92.16%	93.17%
		ResNet50(x2)	NEG	94.05%	91.81%	96.91%	94.29%
			DEL		93.84%	86.15%	89.83%
			INS		97.82%	95.69%	96.74%
			macro avg		94.49%	92.91%	93.62%
		ResNet200(x2)	NEG	93.39%	91.23%	95.41%	93.27%
			DEL		94.99%	83.89%	89.10%
			INS		95.69%	97.50%	96.59%
			macro avg		93.97%	92.27%	92.98%

Table S7. HG002 dataset. Different classification result metrics of CSV-Filter in ResNet50, ResNet50(x2), and ResNet200(x2) self-supervised models.

Filter	Type	Model	Classification	Accuracy	Precision	Recall	F1 score
CSV-Filter	Self-supervised	ResNet34	NEG	93.20%	91.56%	96.06%	93.76%
			DEL		93.96%	88.58%	91.19%
			INS		95.31%	92.39%	93.83%
			macro avg		93.61%	92.34%	92.93%
		ResNet50	NEG	93.97%	90.95%	97.65%	94.18%
			DEL		96.35%	84.00%	89.75%
			INS		97.32%	95.92%	96.62%
			macro avg		94.87%	92.52%	93.52%
		ResNet50(x2)	NEG	94.94%	93.24%	96.94%	95.06%
			DEL		96.73%	89.20%	92.81%
			INS		96.34%	96.22%	96.28%
			macro avg		95.44%	94.12%	94.72%
		ResNet200(x2)	NEG	93.65%	91.59%	95.66%	93.58%
			DEL		97.15%	83.46%	89.78%
			INS		94.59%	98.28%	96.40%
			macro avg		94.44%	92.47%	93.26%

Table S8. NA12878 dataset. Different classification result metrics of CSV-Filter in MobileNet_v2, ResNet34, and ResNet50 CNN models.

Filter	Type	Model	Classification	Accuracy	Precise	Recall	F1 score
CSV-Filter	CNN	MobileNet v2	NEG	87.39%	87.97%	91.14%	89.53%
			DEL		85.48%	71.51%	77.87%
			INS		87.67%	94.83%	91.11%
			macro avg		87.04%	85.83%	86.17%
		ResNet34	NEG	87.62%	87.27%	92.21%	89.67%
			DEL		85.88%	71.92%	78.28%
			INS		89.58%	93.28%	91.39%
			macro avg		87.58%	85.80%	86.45%
		ResNet50	NGE	87.78%	88.45%	90.49%	89.46%
			DEL		85.63%	73.22%	78.94%
			INS		88.13%	96.01%	91.90%
			macro avg		87.40%	86.57%	86.77%

Table S9. NA12878 dataset. Different classification result metrics of CSV-Filter in ResNet50, ResNet50(x2), and ResNet200(x2) self-supervised learning models.

Filter	Type	Model	Classification	Accuracy	Precise	Recall	F1 score
CSV-Filter	Self-supervised	ResNet50	NEG	88.29%	89.30%	90.88%	90.08%
			DEL		84.16%	75.45%	79.57%
			INS		89.63%	95.15%	92.31%
			macro avg		87.70%	87.16%	87.32%
		ResNet50(x2)	NEG	87.87%	87.95%	92.16%	90.01%
			DEL		86.47%	72.58%	78.92%
			INS		88.74%	93.73%	91.17%
			macro avg		87.87%	86.16%	86.70%
		ResNet200(x2)	NGE	88.24%	87.92%	92.69%	90.24%
			DEL		87.50%	72.31%	79.19%
			INS		89.40%	94.40%	91.83%
			macro avg		88.27%	86.47%	87.09%

Table S10. HG002 dataset. The filtering performance of CSV-Filter on deletion detection for long reads.

Platform	Caller	Raw			Filtered		
		Recall[1]	Precision[2]	F1 score	Recall	Precision	F1 score
PacBio CLR	cuteSV	97.39%	96.64%	97.02%	97.31%	97.24%	**97.27%**
	PBSV	95.45%	90.97%	93.15%	95.14%	96.83%	**95.98%**
	Sniffles2	94.83%	96.60%	95.71%	94.62%	97.00%	**95.79%**
	SVIM	97.43%	95.28%	96.34%	97.24%	96.21%	**96.72%**
	SVision	96.14%	91.38%	93.70%	95.98%	95.43%	**95.70%**
PacBio HiFi	cuteSV	99.05%	96.74%	97.88%	98.71%	97.07%	**97.89%**
	PBSV	98.64%	91.76%	95.07%	97.55%	95.43%	**96.48%**
	Sniffles2	99.14%	95.13%	97.09%	98.98%	95.58%	**97.25%**
	SVIM	98.71%	94.44%	96.52%	98.55%	95.54%	**97.02%**
	SVision	98.40%	94.08%	96.19%	98.14%	94.67%	**96.38%**
ONT	cuteSV	98.53%	95.57%	97.03%	98.52%	96.23%	**97.36%**
	PBSV	96.69%	85.70%	90.89%	96.24%	88.57%	**92.26%**
	Sniffles2	98.50%	95.72%	97.10%	98.50%	96.14%	**97.31%**
	SVIM	98.67%	94.22%	96.39%	98.66%	94.78%	**96.69%**
	SVision	98.13%	92.44%	95.20%	98.12%	96.13%	**97.12%**

Recall, Precision, and F1 score in SV calling. The read sequences are from PacBio CLR, PacBio HiFi, and ONT of sample HG002. PBSV does not support ONT sequence SV calling.

[1] The proportion of TP numbers to actual positive numbers in the gold standard dataset ($\frac{TP}{TP+FN}$).

[2] The proportion of TP numbers in the detected SVs ($\frac{TP}{TP+FP}$).

Table S11. HG002 dataset. The filtering performance of CSV-Filter on insertion detection for long reads.

Platform	Caller	Raw			Filtered		
		Recall[1]	Precision[2]	F1 score	Recall	Precision	F1 score
PacBio CLR	cuteSV	94.38%	89.09%	91.66%	94.29%	89.30%	**91.72%**
	PBSV	81.45%	83.41%	82.42%	80.80%	89.86%	**85.09%**
	Sniffles2	94.62%	61.51%	74.55%	94.10%	67.11%	**78.34%**
	SVIM	87.62%	93.22%	**90.33%**	87.49%	93.26%	90.28%
	SVision	91.15%	78.76%	84.58%	91.05%	80.04%	**85.19%**
PacBio HiFi	cuteSV	95.99%	94.40%	95.19%	95.98%	94.65%	**95.31%**
	PBSV	77.03%	75.88%	76.45%	76.67%	91.24%	**83.32%**
	Sniffles2	96.86%	91.00%	93.84%	96.85%	91.33%	**94.01%**
	SVIM	95.48%	90.43%	92.83%	95.35%	90.96%	**93.16%**
	SVision	94.58%	88.57%	91.45%	94.52%	89.18%	**91.80%**
ONT	cuteSV	96.54%	93.87%	95.19%	96.44%	94.07%	**95.24%**
	PBSV	81.66%	85.02%	83.31%	80.97%	86.42%	**83.60%**
	Sniffles2	96.15%	92.16%	**94.12%**	96.01%	92.25%	94.09%
	SVIM	93.90%	87.77%	90.73%	93.70%	88.65%	**91.10%**
	SVision	92.50%	72.72%	81.43%	92.41%	73.49%	**81.94%**

Recall, Precision, and F1 score in SV calling. The read sequences are from PacBio CLR, PacBio HiFi, and ONT of sample HG002. PBSV does not support ONT sequence SV calling.

[1] The proportion of TP numbers to actual positive numbers in the gold standard dataset ($\frac{TP}{TP+FN}$).

[2] The proportion of TP numbers in the detected SVs ($\frac{TP}{TP+FP}$).

Table S12. Telomere-to-Telomere assembly of CHM13. The filtering performance of CSV-Filter on deletion detection for long reads.

Datasets	Caller	Raw			Filtered		
		Recall[1]	Precision[2]	F1 score	Recall	Precision	F1 score
PacBio CLR	cuteSV	82.52%	93.13%	85.81%	82.22%	94.93%	**88.17%**
	PBSV	80.93%	83.39%	82.14%	80.76%	93.09%	**86.54%**
	Sniffles2	79.26%	89.28%	83.98%	79.12%	94.83%	**86.37%**
PacBio HiFi	cuteSV	84.80%	91.05%	87.82%	84.79%	91.37%	**87.95%**
	PBSV	79.97%	78.41%	79.18%	79.88%	92.69%	**85.87%**
	Sniffles2	78.54%	82.37%	80.42%	78.23%	93.21%	**85.13%**
ONT	cuteSV	86.87%	89.02%	87.93%	86.72%	90.58%	**88.61%**
	PBSV	86.87%	83.38%	**85.09%**	83.94%	85.72%	84.82%
	Sniffles2	81.85%	82.51%	82.17%	81.67%	88.68%	**85.03%**

Recall, Precision, and F1 score in SV calling. The read sequences are from PacBio CLR, PacBio HiFi, and ONT read alignment files. SV callsets were benchmarked in the high-confidence regions suggested by Dipcall.
[1] The proportion of TP numbers to actual positive numbers in the gold standard dataset ($\frac{TP}{TP+FN}$).
[2] The proportion of TP numbers in the detected SVs ($\frac{TP}{TP+FP}$).

Table S13. Telomere-to-Telomere assembly of CHM13. The filtering performance of CSV-Filter on insertion detection for long reads.

Datasets	Caller	Raw			Filtered		
		Recall[1]	Precision[2]	F1 score	Recall	Precision	F1 score
PacBio CLR	cuteSV	77.92%	91.21%	84.10%	77.81%	92.62%	**84.64%**
	PBSV	67.31%	78.21%	72.31%	67.14%	87.46%	**75.94%**
	Sniffles2	62.22%	89.63%	73.86%	62.07%	95.97%	**75.53%**
PacBio HiFi	cuteSV	81.40%	92.26%	**86.56%**	81.21%	92.54%	86.47%
	PBSV	62.52%	78.23%	69.46%	62.31%	92.24%	**74.65%**
	Sniffles2	59.18%	85.91%	69.98%	59.01%	96.88%	**73.46%**
ONT	cuteSV	82.24%	90.82%	86.32%	82.16%	92.50%	**87.05%**
	PBSV	65.53%	79.63%	71.90%	65.32%	87.19%	**74.62%**
	Sniffles2	63.18%	80.80%	70.86%	63.12%	93.87%	**75.49%**

Recall, Precision, and F1 score in SV calling. The read sequences are from PacBio CLR, PacBio HiFi, and ONT read alignment files. SV callsets were benchmarked in the high-confidence regions suggested by Dipcall.

[1] The proportion of TP numbers to actual positive numbers in the gold standard dataset ($\frac{TP}{TP+FN}$).

[2] The proportion of TP numbers in the detected SVs ($\frac{TP}{TP+FP}$).

Table S14. HG002 dataset. The filtering performance of DeepSVFilter and CSV-Filter on deletion detection for short reads.

Caller	Without filtering				Filter	With filtering			
	Call num[1]	Recall[2]	Precision[3]	F1 score		Call num	Recall	Precision	F1 score
DELLY	1756	32.29%	77.23%	45.46%	CSV-Filter	1441	31.53%	91.88%	**46.95%**
					DeepSVFilter	1195	26.34%	73.15%	38.73%
LUMPY	2992	57.84%	81.14%	67.51%	CSV-Filter	2951	57.73%	82.10%	**67.86%**
					DeepSVFilter	2159	43.32%	84.25%	57.22%
Manta	3242	72.20%	93.60%	81.47%	CSV-Filter	3206	71.80%	94.04%	**81.43%**
					DeepSVFilter	2798	63.73%	95.64%	76.49%
SvABA	2202	34.00%	64.80%	44.72%	CSV-Filter	1875	33.51%	89.30%	**48.71%**
					DeepSVFilter	1338	31.27%	98.13%	47.43%
Cue[4]	128	92.54%	96.88%	94.66%	CSV-Filter	127	92.54%	97.64%	**95.02%**
					DeepSVFilter	88	64.93%	98.86%	78.39%

Recall, Precision, and F1 score in SV calling. The read sequences are from Illumina of sample HG002.
[1] The SVs number detected by SV detection tools.
[2] The proportion of TP numbers to actual positive numbers in the gold standard dataset ($\frac{TP}{TP+FN}$).
[3] The proportion of TP numbers in the detected SVs ($\frac{TP}{TP+FP}$).
[4] Cue is designed for detecting long SVs [11], and the results in the table are for the SVs longer than 5,000 bp.

Table S15. HG002 dataset. Accuracy, micro-average values of Precision, Recall, and F1 score, as well as F1 score for NEG, DEL, and INS of CSV-Filter with and without mixed precision on different models.

Filter	Type	Model	Accuracy	Precision	Recall	F1 score	F1 score		
							NEG[1]	DEL[2]	INS[3]
CSV-Filter	CNN	MobileNet v2	93.07%	94.30%	91.39%	92.55%	96.46%	88.12%	93.09%
without		ResNet34	93.74%	94.40%	92.39%	93.23%	96.89%	88.89%	93.90%
mixed precision		ResNet50	92.94%	94.05%	91.22%	92.32%	96.62%	87.32%	93.03%
CSV-Filter	CNN	MobileNet v2	92.84%	93.89%	91.31%	92.39%	95.05%	88.95%	93.19%
with		ResNet34	93.20%	93.61%	92.34%	92.93%	93.83%	91.19%	93.76%
mixed precision		ResNet50	93.61%	94.60%	92.16%	93.17%	96.16%	89.57%	93.79%

[1] F1 score for non-structural variants.
[2] F1 score for deletion variants.
[3] F1 score for insertion variants.

Table S16. NA12878 dataset. Accuracy, micro-average values of Precision, Recall, and F1 score, as well as F1 score for NEG, DEL, and INS of CSV-Filter with multi-channel image and grayscale image on different models.

Filter	Type	Model	Accuracy	Precision	Recall	F1 score	F1 score		
							NEG[1]	DEL[2]	INS[3]
CSV-Filter	CNN	MobileNet v2	80.11%	78.24%	75.68%	76.70%	88.36%	70.63%	71.12%
multi		ResNet34	80.58%	78.66%	76.33%	77.27%	88.76%	71.54%	71.53%
channels		ResNet50	80.09%	77.67%	76.64%	77.10%	88.32%	71.60%	71.37%
CSV-Filter	CNN	MobileNet v2	87.39%	87.04%	85.83%	86.17%	89.53%	77.87%	91.11%
grayscale		ResNet34	87.62%	87.58%	85.80%	86.45%	89.67%	78.28%	91.39%
		ResNet50	87.78%	87.40%	86.57%	86.77%	89.46%	78.94%	91.90%

[1] F1 score for non-structural variants.
[2] F1 score for deletion variants.
[3] F1 score for insertion variants.

Table S17. NA12878 dataset. The filter performance CSV-Filter on the SV results generated by structural variant detection tools PBSV and Sniffles2 with trained self-Supervised ResNet50, ResNet50(x2), and ResNet200(x2) models.

SV type	Caller	Models	Raw			Filtered		
			Call num[1]	Recall[2]	Precision[3]	Call num	Recall	Precision
INS	PBSV	resnet50	5310	2.34%	2.52%	1338	2.10%	8.97%
		resnet50x2				1563	2.32%	8.51%
		resnet200x2				1646	2.25%	7.84%
	Sniffles2	resnet50	9427	7.29%	4.42%	6653	7.04%	6.06%
		resnet50x2				6652	7.11%	6.12%
		resnet200x2				6737	7.20%	6.12%
	NGMLR	resnet50	9745	45.88%	26.95%	7464	44.15%	32.93%
	+	resnet50x2				7781	45.11%	33.18%
	Sniffles2	resnet200x2				7822	45.28%	33.14%
DEL	PBSV	resnet50	8308	52.19%	23.64%	5291	43.90%	31.22%
		resnet50x2				5038	41.62%	31.08%
		resnet200x2				4884	40.21%	30.98%
	Sniffles2	resnet50	6118	54.05%	33.25%	5028	51.82%	38.78%
		resnet50x2				5010	51.47%	38.66%
		resnet200x2				5028	51.74%	38.72%
	NGMLR	resnet50	8016	67.98%	31.91%	5772	64.58%	42.10%
	+	resnet50x2				5775	64.15%	41.80%
	Sniffles2	resnet200x2				5769	64.60%	42.14%

The SV detection tools take BAM files generated by BLASR (Version 1.3.2) as the default input. In the Sniffles2 comparative experiment, the alignment tool used to generate BAM files is NGMLR (Version 0.2.7).

[1] The SVs number detected by SV detection tools.

[2] The proportion of TP numbers to actual positive numbers in the gold standard dataset ($\frac{TP}{TP+FN}$).

[3] The proportion of TP numbers in the detected SVs ($\frac{TP}{TP+FP}$).

8. SUPPLEMENTARY NOTES

A. Software versions

We used the following versions of software tools in our benchmarks:

Aligner:

- minimap2 2.28-r1209
- pbmm2 1.13.1
- NGMLR 0.2.7
- BWA-MEM 0.7.17-r1188

SV detect/filter tools:

- PBSV 2.9.0
- Sniffles2 2.0.7
- SVIM 2.0.0
- cuteSV 2.0.3
- SVision 1.3.8
- Dipcall 0.3
- DELLY 1.1.5
- LUMPY 0.2.13
- Manta 1.6.0
- Cue 0.7.0
- SvABA 1.2.0
- samtools 1.5
- bedtools 2.26.0
- sambaba 0.6.7-pre1
- DeepSVFilter (Date: Nov 10, 2020)

Evaluation tool:

- Truvari 3.5.0

B. Execution parameters

B.1. Aligner

In this section we report the command lines of the aligners we used in our experimental evaluation.

minimap2. We ran minimap2 with default parameters following recommended workflow:

Align clr/hifi/ont reads with 8 threads and sort the bam file with samtools:

```
$ minimap2 -t 8 --MD -Y -L -a -x map-clr/hifi/ont reference.fa input.fastq \
  | samtools sort -o output.bam
```

pbmm2. pbmm2 is used for PacBio CLR/HiFi data.

Align reads with 4 threads and sort the bam file:

```
$ pbmm2 align -j 8 reference.fa input.fastq output.bam --preset SUBREAD \
        --sort
```

NGMLR. We combined NGMLR with Sniffles2 to get better SV detection results on sample NA12878. We first ran NGMLR to get alignment files:

(1) Do the alignment with 8 threads:
```
$ ngmlr -t 8 -r reference.fa -q input.fastq -o output.sam
```

(2) Convert sam filte to bam file with 8 threads:
```
$ samtools view -@ 8 -b output.sam > output.bam
```

(3) Sort & index bam file with 8 threads:
```
$ samtools sort -@ 8 output.bam -o output_sorted.bam
$ samtools index -@ 8 output_sorted.bam
```

picard. We use picard to add read group (RG) identifiers in the header of the ONT alignment file.

(1) Download picard:
```
$ wget https://github.com/broadinstitute/picard/releases/download \
       /2.26.10/picard.jar
```

(2) Add RG identifiers in the header of the ONT alignment file:
```
$ java -jar picard.jar AddOrReplaceReadGroups \
       I=input.sorted.bam \
       O=output.sorted.withRG.bam \
       RGID=1 \
       RGLB=lib1 \
       RGPL=ONT \
       RGPU=unit1 \
       RGSM=HG002
```

BWA-MEM. We ran BWA-MEM to align second-generation data:

(1) Index the reference genome:
```
$ bwa index reference.fa
```

(2) Align second-generation data with 8 thread:
```
$ bwa mem -t 8 reference.fa input_1.fastq input_2.fastq > output.sam
```

(3) Transfer the SAM file to the BAM file:
```
$ samtools view -Sb output.sam > output.bam
```

(4) Sort the BAM file with 8 threads:
```
$ samtools sort -@ 8 output.bam -o output.sorted.bam
```

(5) Index the BAM file:
```
$ samtools index output.sorted.bam
```

B.2. SV detect/filter tools

In this section we report the command lines of the SV detect/filter tools we used in our experimental evaluation.

PBSV. We ran PBSV with default parameters following recommended workflow:

(1) Discover structural variant features:
```
$ pbsv discover input.bam input.svsig.gz
```

(2) Call structural variants and assigning genotypes:
```
$ pbsv call reference.fa input.svsig.gz pbsv_output.vcf
```

Sniffles2. We ran Sniffles2 with default parameters following recommended workflow:

Generate a VCF (Variant Call Format) file containing insertion and deletion structural variants:
```
$ sniffles -input input.bam -vcf output.vcf -call insert -call delete
```

SVIM. We ran SVIM with default parameters following recommended workflow:

Generate a VCF (Variant Call Format) file:
```
$ svim alignment -i input.bam -r reference.fa -o output_folder
```

cuteSV. We ran cuteSV with default parameters following recommended workflow:

Generate a VCF (Variant Call Format) file with 8 threads to current folder:
```
$ cuteSV -t 8 input.bam reference.fa output.vcf .
```

SVision. We ran SVision with default parameters following recommended workflow:

Generate a VCF (Variant Call Format) file with 8 threads:
```
$ SVision -t 8 -o output_folder -b input.bam -m trained_model.ckpt
          -g reference.fa -n sample_name -s 5 -graph -qname
```

Dipcall. We ran Dipcall with default parameters following recommended workflow:

(1) Generate .mak file:
```
$ run-dipcall -t ${threads} -x ${bed} ${prefix} ref.fa hp1.fa hp2.fa \
              > ${prefix}.mak
```

(2) Run .mak file:
```
$ make -j2 -f ${prefix}.mak
```

DELLY. Usage requirements: only for second-generation sequencing data, BAM files must be sorted, indexed, and have duplicates marked. The reference genome must be indexed.

(1) Sort BAM file:
```
$ samtools sort -o input_sorted.bam input.bam
```

(2) Duplicates mark BAM file: filter & remove duplicates
```
$ samtools view -h -@ 2 -b -q 1 -F 256 input_sorted.bam \
                > input_sorted_filtered.bam
$ sambamba markdup -r -p -t 2 -tmpdir=./ input_sorted_filtered.bam \
                   input_sorted_markdup.bam
```

(3) Index BAM file:
```
$ samtools input_sorted_markdup.bam
```

(4) Generate bcf file:
```
$ ./delly_v1.1.5_linux_x86_64bit call -g reference.fa -o output.bcf \
                                      input_sorted_markdup.bam
```

(5) Convert bcf file to vcf file:
```
$ bcftools view output.bcf > output.vcf
```

LUMPY. LUMPY requires prior preparation of discordant short reads and split-read alignments, followed by the use of encapsulated functions for computation.

(1) Prior preparation:
```
$ samtools view -b -F 1294 input.bam > input.discordants.unsorted.bam
$ samtools view -h input.bam | extractSplitReads_BwaMem -i stdin | \
  samtools view -Sb - > input.splitters.unsorted.bam
$ samtools sort -@ 4 -o input.discordants.bam \
                -T temp input.discordants.unsorted.bam
$ samtools sort input.splitters.unsorted.bam input.splitters.bam
```

(2) Generate vcf file:
```
$ lumpyexpress -B input.bam \
               -S input.splitters.bam \
               -D input.discordants.bam \
               -o output.vcf
```

Manta. Manta should run in its installation directory, and needs configuration first.

(1) Configuration:
```
$ configMant.py -bam input.bam \
                -referenceFasta reference.fa/fasta \
                -runDir .
```

(2) Generate vcf file:
```
$ python runWorkflow.py
```

(3) Unzip:
```
$ gzip -d output.vcf.gz
```

Cue. Cue first needs to configure two YAML files: one for the data (data_config.yaml) and one for the model (model_config.yaml).

(1) Configure the data_config.yaml:
```
bam: /path/to/your/alignment.bam
fai: /path/to/your/reference.fasta.fai
chr_names: [""] (optional)
bed: /path/to/your/ground_truth.bed (optional)
interval_size: 1000 (optional)
step_size: 500 (optional)
report_dir: /path/to/report (optional)
blacklist_bed: /path/to/blacklist.bed (optional)
refine_disable: false (optional)
min_qual_score: 30 (optional)
min_sv_len: 50 (optional)
```

(2) Configure the model_config.yaml:
```
python runWorkflow.py
model_path: /path/to/pretrained_model.pth (optional)
gpu_ids: [0] (optional)
n_jobs_per_gpu: 1 (optional, default 1)
n_cpus: 1 (optional)
batch_size: 32 (optional)
logging_level: INFO (optional)
```

(3) Navigate to the root directory of the Cue framework in the command line, then run the following command:
```
$ export PYTHONPATH=${PYTHONPATH}:/home/usr/cue
$ python engine/call.py --data_config /path/to/data_config.yaml \
                        --model_config /path/to/model_config.yaml
```

SvABA. SvABA needs to be run in the build folder located in its root directory, using the executable file ./svaba.

Generate vcf file:
```
$ ./svaba run -G reference.fa -t input.bam
```

DeepSVFilter. The files downloaded from DeepSVFilter contain a pre-trained MobileNet model. During the execution, we performed vcf2bed, preprocess, and predict operations separately, and all operations were run with default settings

(1) Use vcf2bed to convert SV vcf file to bed file:
```
$ vcf2bed --sv_type DEL or DUP \
          --vcf_file input.vcf \
          --tool_name delly,manta,lumpy or giab \
          --exclude exclude bed file (optional, default NULL) \
          --length SV length (optional, default 100) \
          --bed_file output.bed
```

(2) Use preprocess to generate SV images for candidate SVs:

```
$ DeepSVFilter preprocess --sv_type DEL or DUP \
                          --bam_path input.bam \
                          --bed_path input.bed \
                          --patch_size 224 or 299 \
                          --mean_insert_siz default (optional) \
                          --sd_insert_size default (optional) \
                          --output_imgs_dir SV image path file
```

(3) Use predict with pre-trained MobileNet model to make predictions for candidate SVs:

```
$ DeepSVFilter predict --sv_type DEL or DUP \
                       --checkpoint_dir checkpoint folder \
                       --test_file SV image path file \
                       --test_result_dir SV filtering results \
                       --use_gpu 1 = GPU, 0 = CPU (optional, default 0) \
                       --gpu_idx idx (optional, default 0) \
                       --gpu_mem usage (0 to 1) (optional, default 0.5) \
                       --model M1
```

B.3. Evaluation tool

In this section we report the command lines of the Evaluation tool we used in our experimental evaluation.

Truvari. We ran Truvari with parameters following followed those in the review "Trade-offs in alignment and assembly-based methods for structural variant detection with long-read sequencing data":

(1) Sort vcf file chromesome index:

```
$ sort-vcf input.vcf > input.sorted.vcf
```

(2) Unzip and index vcf file:

```
$ bgzip -c input.sorted.vcf > input.sorted.vcf.gz
$ tabix -p vcf input.sorted.vcf.gz
```

(3) Compare the input vcf file to base vcf file:

```
$ truvari bench -passonly -p 0 -P 0.5 -r 500 -O 0 \
                -f reference.fa \
                -c input.sorted.vcf.gz \
                -b base.vcf.gz \
                --include high_confidence_region.bed \
                -o output_file
```

The parameters set in Truvari mean:

- Parameter p controls the minimum allele sequence similarity used to identify two SV calls as the same. The parameter p is set to 0 to disable SV sequence comparison.
- Parameter P corresponds to the minimum allele size similarity for comparing SVs. The parameter P is set to 0.5, requiring that the size similarity of the two variants being compared must be at least 50%.
- Parameter r limits the threshold for the maximum reference position difference between SVs. The parameter r is set to 500, allowing a positional deviation of up to 500 bp in the reference genome.
- Parameter O determines the threshold for the minimum mutual overlap ratio between the base and comparison calls. The parameter O is set to 0 to allow breakpoint shifts for deletions.

REFERENCES

1. J. M. Zook, N. F. Hansen, N. D. Olson, *et al.*, "A robust benchmark for detection of germline large deletions and insertions," Nat. biotechnology **38**, 1347–1355 (2020).
2. J. M. Zook, B. Chapman, J. Wang, *et al.*, "Integrating human sequence data sets provides a resource of benchmark snp and indel genotype calls," Nat. biotechnology **32**, 246–251 (2014).
3. P. H. Sudmant, T. Rausch, E. J. Gardner, *et al.*, "An integrated map of structural variation in 2,504 human genomes," Nature **526**, 75–81 (2015).
4. J. Deng, W. Dong, R. Socher, *et al.*, "Imagenet: A large-scale hierarchical image database," in *2009 IEEE conference on computer vision and pattern recognition,* (Ieee, 2009), pp. 248–255.
5. A. C. English, W. J. Salerno, and J. G. Reid, "Pbhoney: identifying genomic variants via long-read discordance and interrupted mapping," BMC bioinformatics **15**, 1–7 (2014).
6. "Readme.txt for na12878_pacbio_mtsinai data," (2015).
7. M. J. Chaisson, J. Huddleston, M. Y. Dennis, *et al.*, "Resolving the complexity of the human genome using single-molecule sequencing," Nature **517**, 608–611 (2015).
8. M. A. Eberle, E. Fritzilas, P. Krusche, *et al.*, "A reference data set of 5.4 million phased human variants validated by genetic inheritance from sequencing a three-generation 17-member pedigree," Genome research **27**, 157–164 (2017).
9. H. Li, J. M. Bloom, Y. Farjoun, *et al.*, "A synthetic-diploid benchmark for accurate variant-calling evaluation," Nat. methods **15**, 595–597 (2018).
10. J. M. Zook, D. Catoe, J. McDaniel, *et al.*, "Extensive sequencing of seven human genomes to characterize benchmark reference materials," Sci. data **3**, 1–26 (2016).
11. V. Popic, C. Rohlicek, F. Cunial, *et al.*, "Cue: a deep-learning framework for structural variant discovery and genotyping," Nat. methods **20**, 559–568 (2023).
